# Successful intergeneric transfer of a major apple scab resistance gene (*Rvi6*) from apple to pear and precise comparison of the downstream molecular mechanisms of this resistance in both species

**DOI:** 10.1186/s12864-021-08157-1

**Published:** 2021-11-22

**Authors:** L. Perchepied, E. Chevreau, E. Ravon, S. Gaillard, S. Pelletier, M. Bahut, P. Berthelot, R. Cournol, H. J. Schouten, E. Vergne

**Affiliations:** 1 Univ Angers, Institut Agro, INRAE, IRHS, SFR QUASAV, F-49000 Angers, France; 2 Univ Angers, SFR QUASAV, F-49000 Angers, France; 3 Wageningen Univ & Res, Plant Breeding, NL-6700 Wageningen, AJ Netherlands

**Keywords:** Apple, Intergeneric, Pear, Rvi6, Scab, Transcriptomics, Transgenesis

## Abstract

**Background:**

Scab is the most important fungal disease of apple and pear. Apple (*Malus x domestica* Borkh.) and European pear (*Pyrus communis* L.) are genetically related but they are hosts of two different fungal species: *Venturia inaequalis* for apple and *V. pyrina* for European pear. The apple/*V. inaequalis* pathosystem is quite well known, whereas knowledge about the pear/*V. pyrina* pathosystem is still limited. The aim of our study was to analyse the mode of action of a major resistance gene of apple (*Rvi6*) in transgenic apple and pear plants interacting with the two scab species (*V. inaequalis* and *V. pyrina*), in order to determine the degree of functional transferability between the two pathosystems.

**Results:**

Transgenic pear clones constitutively expressing the *Rvi6* gene from apple were compared to a scab transgenic apple clone carrying the same construct. After inoculation in greenhouse with *V. pyrina*, strong defense reactions and very limited sporulation were observed on all transgenic pear clones tested. Microscopic observations revealed frequent aborted conidiophores in the *Rvi6* transgenic pear / *V. pyrina* interaction. The macro- and microscopic observations were very comparable to the *Rvi6* apple / *V. inaequalis* interaction. However, this resistance in pear proved variable according to the strain of *V. pyrina*, and one of the strains tested overcame the resistance of most of the transgenic pear clones. Comparative transcriptomic analyses of apple and pear resistant interactions with *V. inaequalis* and *V. pyrina*, respectively, revealed different cascades of molecular mechanisms downstream of the pathogen recognition by *Rvi6* in the two species. Signal transduction was triggered in both species with calcium (and G-proteins in pear) and interconnected hormonal signaling (jasmonic acid in pear, auxins in apple and brassinosteroids in both species), without involvement of salicylic acid. This led to the induction of defense responses such as a remodeling of primary and secondary cell wall, lipids biosynthesis (galactolipids in apple and cutin and cuticular waxes in pear), systemic acquired resistance signal generation (in apple) or perception in distal tissues (in pear), and the biosynthesis of phenylpropanoids (flavonoids in apple but also lignin in pear).

**Conclusion:**

This study is the first example of a successful intergeneric transfer of a resistance gene among *Rosaceae*, with a resistance gene functioning towards another species of pathogen.

**Supplementary Information:**

The online version contains supplementary material available at 10.1186/s12864-021-08157-1.

## Background

Apple (*Malus domestica* Borkh.) and European pear (*Pyrus communis* L.) are two closely related species of great economic importance for fruit production. A range of pests and diseases attacks both species and their production require a high number of treatments. Scab, caused by *Venturia* species, is the most damaging fungal disease of both fruit species in all temperate countries. This disease causes necrotic lesions on leaves and fruits, which decrease the tree vigor and reduce fruit quality, which make fruits unsuitable for fresh market sales. Chemical scab control under oceanic climates usually requires spraying up to 20 treatments per year and the development of alternative production systems (integrated protection, organic farming) reduce only partially the number of treatments [1]. A sustainable approach is the breeding of new varieties carrying durable resistance toward this disease. To achieve this goal, a better understanding of the function of major resistance genes and downstream defenses is needed.

Apple and European pears are hosts of two different fungal species: *Venturia inaequalis* for apple and *V. pyrina* (formerly named *V. pirina* [2]) for European pear. A long history of association between host and pathogen permitted their coevolution, which led to a narrow host spectrum for each *Venturia* species (i.e. genus specific) [3]. The level of genetic knowledge of the two pathosystems is very different. In the case of apple scab, numerous major resistance genes (R genes) and quantitative trait loci (QTLs) have been identified [4] and apple/*V. inaequalis* was one of the first plant pathosystem with good evidence for gene-for-gene interactions [5]. On the contrary, knowledge about the pear/*V. pyrina* pathosystem is limited. *V. pyrina* presents at least five physiological races, which were found to have a very narrow range of pathogenicity [6]. So far, only one R gene and several QTLs have been identified [7].

*Rvi6* (formerly *HcrVf2*) is a major scab R gene which has been widely used in apple breeding programs. It is the first resistance gene of apple which has been isolated [8]. It encodes a receptor-like protein (RLP) gene containing an extracellular leucine-rich repeat and a putative transmembrane domain, resembling those of the *Cf9* tomato gene of *Cladosporium fulvum* resistance [9]. Transgenic apple lines expressing *Rvi6* under various promoter sequences present a strong resistance to several Rvi6-avirulent scab strains [10]. Genome-wide molecular analyses of the plant responses to scab have rarely been performed and only on apple host interactions. Subtractive hybridization [11, 12] and cDNA-AFLP [13] led to the identification of a limited set of differentially expressed genes in *Rvi6* natural resistant ‘Florina’ variety (scab inoculated ‘Florina’ versus mock, [12]), or in *Rvi6* resistant transgenic ‘Gala’ lines (*Rvi6* transgenic ‘Gala’ versus non-transformed ‘Gala’, after scab inoculation, [11]; *Rvi6* transgenic ‘Gala’ before versus post scab inoculation, [13]). RNA-seq identified five candidate genes putatively involved in the ontogenic scab resistance of apple [14]. In addition, nuclear proteome analysis identified 13 proteins with differential expression patterns among varying scab resistance ‘Antonovka’ accessions [15]. Therefore, in-depth knowledge of transcriptional patterns and gene functions involved in apple and pear scab resistance is still needed.

Plant immune receptors are the initial key step for recognition of invading pathogens and signalization of plant efficient defense mechanisms. Engineering plants via transfer of such R genes has the potential to increase disease resistance in many crops. Many R genes have now been shown to maintain their function after transfer to other plant species (reviewed in [16, 17]). In most cases, these transfers proved successful inside the *Solaneaceae* or the *Poaceae* families but efficient transfers have also been obtained interfamily or even across the monocot and dicot clades. Several classes of R genes have been successfully transferred between plant species: receptor kinase (RK), RLP, nucleotide binding leucine-rich repeat (NLR). Transfer of R genes acting in a gene-for-gene manner to another pathosystem implies two elements: 1) similarity of pathogen effectors recognized by the R gene and 2) sufficient conservation of downstream signaling pathway leading to efficient defense responses. To our knowledge, transfer of R genes between different pathosystems in the *Rosaceae* family has never been reported.

The objectives of our study were: 1) the functional transfer of the apple scab *Rvi6* gene to the pear/*V. pyrina* pathosystem, 2) the molecular dissection of *Rvi6*-mediated defense responses in the two pathosystems.

## Results and discussion

### Efficient production of pear transgenic lines

The binary plasmid pMF1-pMdRbc1.6-Vf2-tMdRbc [10] containing the *Rvi6* coding sequence and the regulatory promoter and terminator sequences from the apple *Rubisco* gene was used to transform the ‘Conference’ pear variety. In total, 78 kanamycin resistant lines were produced in a single transformation experiment using 650 ‘Conference’ leaf explants, reaching a rate of transformation of 12%. This rate is in the higher range of efficiency of most reports of pear transformation [7]. A sample of 30 lines was checked for ploidy level by flow cytometry. Three tetraploid lines and one chimeric (2n/4n) line were discarded. Polymerase chain reaction (PCR) analysis confirmed the presence of the *Rvi6* transgene and the absence of *Agrobacterium* contamination in the remaining 26 lines. A sample of eight transgenic lines were rooted and acclimatized for scab inoculation in greenhouse, of which functional analyze is available in Fig. S1.

**Fig. 1:**
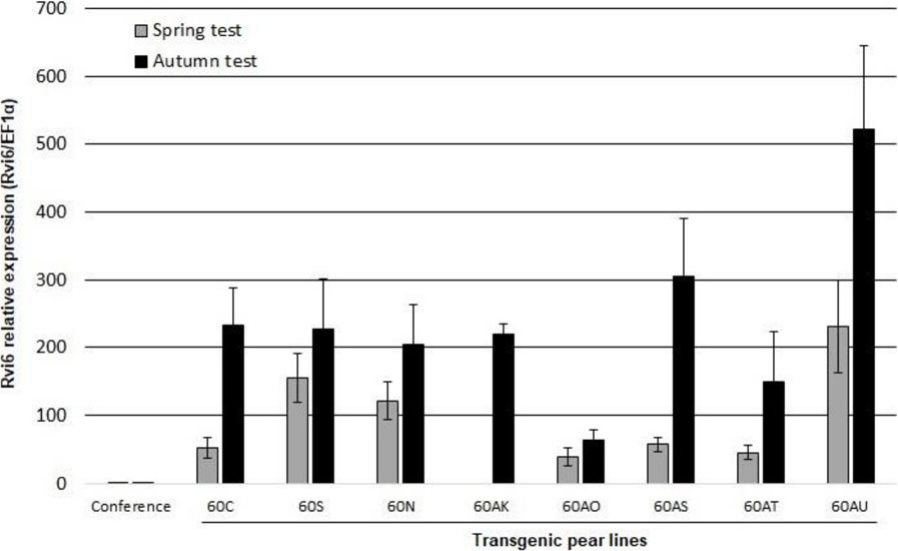
Level of expression of the transgene *Rvi6* in transgenic pear lines. Bars are the mean of three independent replicates, error bars indicate confidence intervals at α = 0.05. Normalization was done with the reference gene *EF1α* and the non-transgenic genotype ‘Conference’ was used as a calibrator

Quantitative polymerase chain reaction (Q-PCR) analyses were performed on leaf samples of these lines at two separate times (Spring and Autumn) to evaluate the level of expression of the transgene *Rvi6*. Results in Fig. 1 indicate a large variability of relative expression of the transgene among the transgenic lines, expression levels are 50 to 500 times greater than a background level in ‘Conference’, which does not possess *Rvi6*. Similarly, Joshi et al. [10] observed a wide variation of expression among apple transgenic lines expressing *Rvi6* controlled by the same apple small subunit rubisco gene promoter (*MdRbc)* (57–163 compared to the natural expression level of this resistance gene in the apple cultivar ‘Santana’ that obtained the *Rvi6* gene by means of conventional breeding), that was not correlated with the copy number of the transgene. In our results, the expression levels measured in Spring were generally lower than those measured in Autumn, but a consistent ranking of the lines was obtained in the two assays. The clone 60 AU appeared as the highest expressing line of *Rvi6* at both sampling dates and was therefore chosen for subsequent transcriptomic analyses.

### High level of *V. pyrina* resistance in pear transgenic lines but possible breakdown with some *V. pyrina strains*

**Fig. 2:**
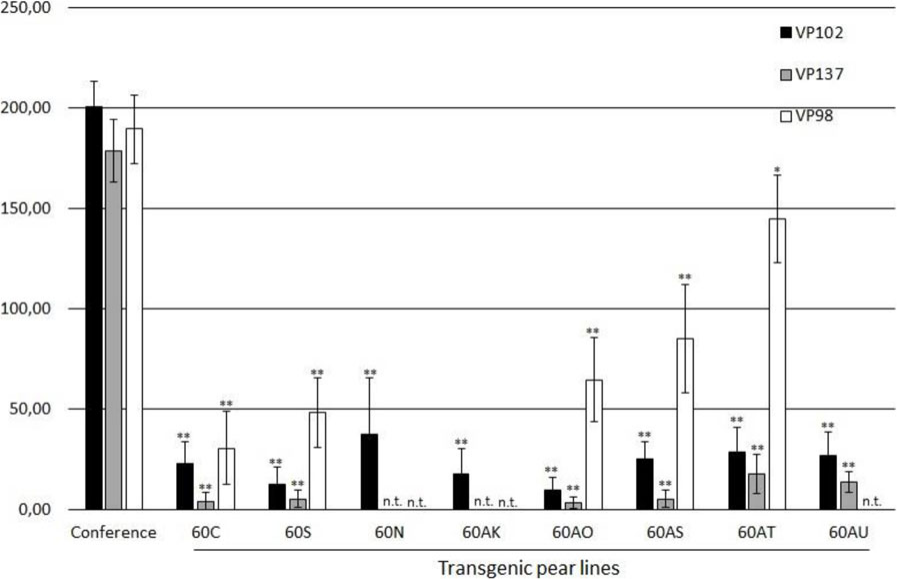
Scab susceptibility of transgenic pear lines inoculated with three different *V. pyrina* strains. AUDPC based on sporulation scores at 14, 21, 28, 35 and 42 days after inoculation with three different *V. pyrina* strains (VP98, VP102, VP137) on a series of transgenic Conference genotypes that received the *Rvi6* gene from apple by means of stable transformation. Bars are the mean of 13 to 25 shoots, error bars indicate confidence intervals at α = 0.05

A scab inoculation test was performed on eight independent pear transgenic lines carrying the *Rvi6* transgene, with 7 to 25 shoots inoculated by line/strain pair, as biological repeats. The area under the disease progress curve (AUDPC) based on sporulation scores at 14, 21, 28, 35 and 42 days after inoculation summarizes the results (Fig. 2). The three strains of *V. pyrina* caused typical severe scab (100% of class 4 symptoms, Table S1) on the non-transgenic ‘Conference’ (susceptible control). Very strong resistance was observed in the seven transgenic lines challenged with strain VP 137, with only 2% of the plants in susceptible class 3b (clear sporulating, chlorotic and necrotic lesions). All the tested transgenic lines were also clearly resistant to strain VP 102, with only 6% of the plants in susceptible class 3b. However, various levels of susceptibility were observed among transgenic pear lines inoculated with strain VP 98. In total, 57% of the plants from all transgenic lines produced susceptible symptoms of classes 3b or 4. Even though the AUDPC of all transgenic lines was significantly lower than ‘Conference’, this indicates a partial breakdown of *Rvi6* resistance in several transgenic pear lines.

These results are very similar to the results of Joshi et al. [10] who tested the scab resistance of apple transgenic lines expressing the same construct. They observed total resistance of the transgenic apple lines towards four isolates of *V. inaequalis* avirulent on *Rvi6*-based resistant cultivars, but this resistance was overcome by the isolate EU-D42, virulent on *Rvi6*-based resistant cultivars. Little is known so far about the effectors of *Venturia* species and the basis of their host specificity. Whole genome sequencing allowed a comparative analysis of the predicted secretomes of *V. inaequalis* and *V. pyrina* [18]. This led to the identification of many candidate effector genes or gene families, some of them being unique to *V. inaequalis* or to *V. pyrina* isolates. Recently, the *AvrRvi6* from *V. inaequalis* has been identified as a 93 amino acid protein containing 6 cysteines [19], but no precise homologous has yet been identified in *V. pyrina*. Our findings indicate that the apple transgene *Rvi6* expressed in pear probably recognizes avirulence effectors similar to *AvrRvi6,* secreted by *V. pyrina*, and that some *V. pyrina* strains possess virulence factors leading to *Rvi6* resistance breakdown.

No significant correlation could be found among the transgenic pear lines between the expression level of *Rvi6* and the degree of resistance or the class of symptoms.

### Variable expression of resistance symptoms

**Fig. 3:**
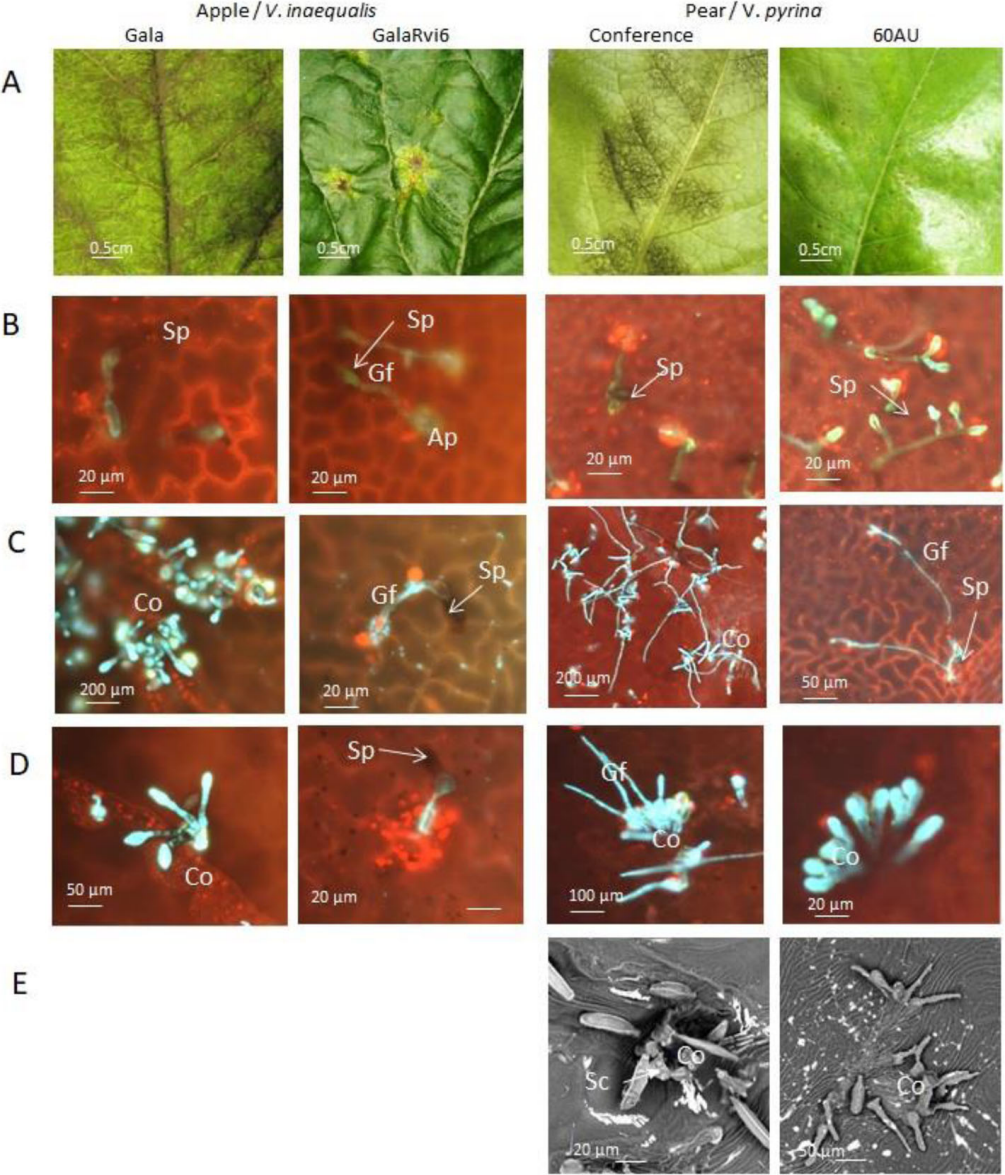
Macro- and microscopic observations of apple and pear susceptible and resistant interactions. (**A**) binocular observations 42 days after inoculatione; (**B**) wide field fluorescence observations 3 days after inoculation; (**C**) wide field fluorescence observations 7 days after inoculation (**D**) wide field fluorescence observations 14 days after inoculation; (**E**) SEM observation 19 days after inoculation. Ap: appressorium, C: conidia, Co: conidiophore, Gf: germination filament, Sc: scar, Sp: spore

At the macroscopic level (Fig. 3A), susceptible interactions (apple: Gala/*V. inaequalis* and pear: Conference/*V. pyrina*) led to a strong sporulation appearing on the upper side of the leaves, but also in the case of pear on the lower side of the leaves as well as on the shoots, as observed previously on ‘Conference’ [20]. At the microscopic level, the kinetics of fungal development was very similar in apple and pear susceptible interactions. Conidia germination and formation of appressoria were achieved three days after inoculation (Fig. 3B). Seven days after inoculation, numerous conidiophores were formed and released conidia (Fig. 3C, D), and the intensity of sporulation was indicated by the number of scars (Fig. 3E).

At the macroscopic level (Fig. 3A), resistant interactions (GalaRvi6/*V. inaequalis* and 60 AU/*V. pyrina*) presented varied types of symptoms. In addition to pin-points, chlorotic and necrotic lesions, with or without leaf crinkling, were frequently observed in apple as well as in pear. At the microscopic level, apple resistance was characterized by short germination filaments. In addition, infected sites surrounded by a ring of red autofluorescent cells around the appressoria were frequently observed (probably due to accumulation of phenolic compounds). Subcuticular stroma was visible, but no conidiophores were observed (Fig. 3B, C and D). In pear, long branched germination filaments were frequent (Fig. 3C) and many aborted conidiophores without conidia emission were observed (Fig. 3D and E).

The kinetics of establishment of the susceptible interactions agrees with previous reports on *V. inaequalis* [21, 22] and *V. pyrina* [20]. Similarly, the large range of resistance symptoms, from pin-points typical of hypersensitive reaction (HR) to chlorotic lesions with occasional very slight sporulation, has been frequently observed in apple genotypes carrying the *Rvi6* gene, provided by conventional breeding [23] as well as on pear cultivars carrying partial to strong resistance [24]. The microscopic observations fit with the histological description of resistance symptoms of class 1, 2 or 3a in *Rvi6* apple genotypes [21].

Based on these findings, we decided to perform the transcriptomic study at 8, 24 and 72 h post inoculation (hpi), in order to cover the period of establishment of the first intimate contact between fungal and plant cells.

### Common and specific patterns of gene expression modulation during the first steps of *Rvi6*-induced resistance in apple versus pear

Differential expressed genes (DEGs) were analysed by comparing transcript abundance in leaves between susceptible non-transgenic and resistant *Rvi6* expressing lines, in apple and in pear, at each of the three time-points of the interaction with *V. inaequalis* and *V. pyrina* respectively. In total, 2977 DEGs in apple and 4170 DEGs in pear were identified, which amounts to 9.5% of all apple genes on the apple AryANE v2.0 microarray, and 9.5% of all pear genes on the Pyrus v1.0 microarray. (Table 1).

**Table 1:** Number of differentially expressed genes (DEGs) identified at each of the three time points

	GalaRvi6 versus Gala				60 AU versus Conference			
	0 hpi	8 hpi	24 hpi	72 hpi	0 hpi	8 hpi	24 hpi	72 hpi
Total number of DEGs*	173	1799	823	539	1115	2415	922	273
DEGs in % of all genes on the microarray**	0.55	5.75	2.63	1.72	2.54	5.50	2.10	0.62
% of upregulated DEGs	19.1	56.8	60.9	30.4	46.2	76.3	81.5	49.1
% of downregulated DEGs	80.9	43.2	39.1	69.6	53.8	23.7	18.5	50.9
% of DEGs without TAIR name	2.89	4.84	6.80	5.19	13.9	14.0	10.1	14.3

*: DEGs numbers were calculated using the *p*-adj values ≤0.01 as selection threshold

**: 66792 genes on the apple AriANE 2.0 microarray, 43,906 genes on the Pyrus v1.0 microarray

In apple GalaRvi6 as in pear 60 AU transgenic lines, *Rvi6* is under the control of the strong constitute Rubisco gene promoter. However, the reaction to these constitutive *RVi6* expression is quite different between apple and pear in terms of DEG quantity. Indeed at T0, before scab inoculation, only 173 DEGs were detected between ‘Gala’ and GalaRvi6, among which 81% were downregulated. On the contrary, in pear, 1115 DEGs between ‘Conference’ and 60 AU were detected at T0, among which 74% were specific of this constitutive state. 46% of these DEGs were up-regulated. Using MapMan to map the DEGs TAIR names, we observed that beside protein and RNA metabolisms, the main functional categories represented in this set of DEGs were signaling, cell cycle, transport, stress and development (Fig. S2).

**Fig. 4:**
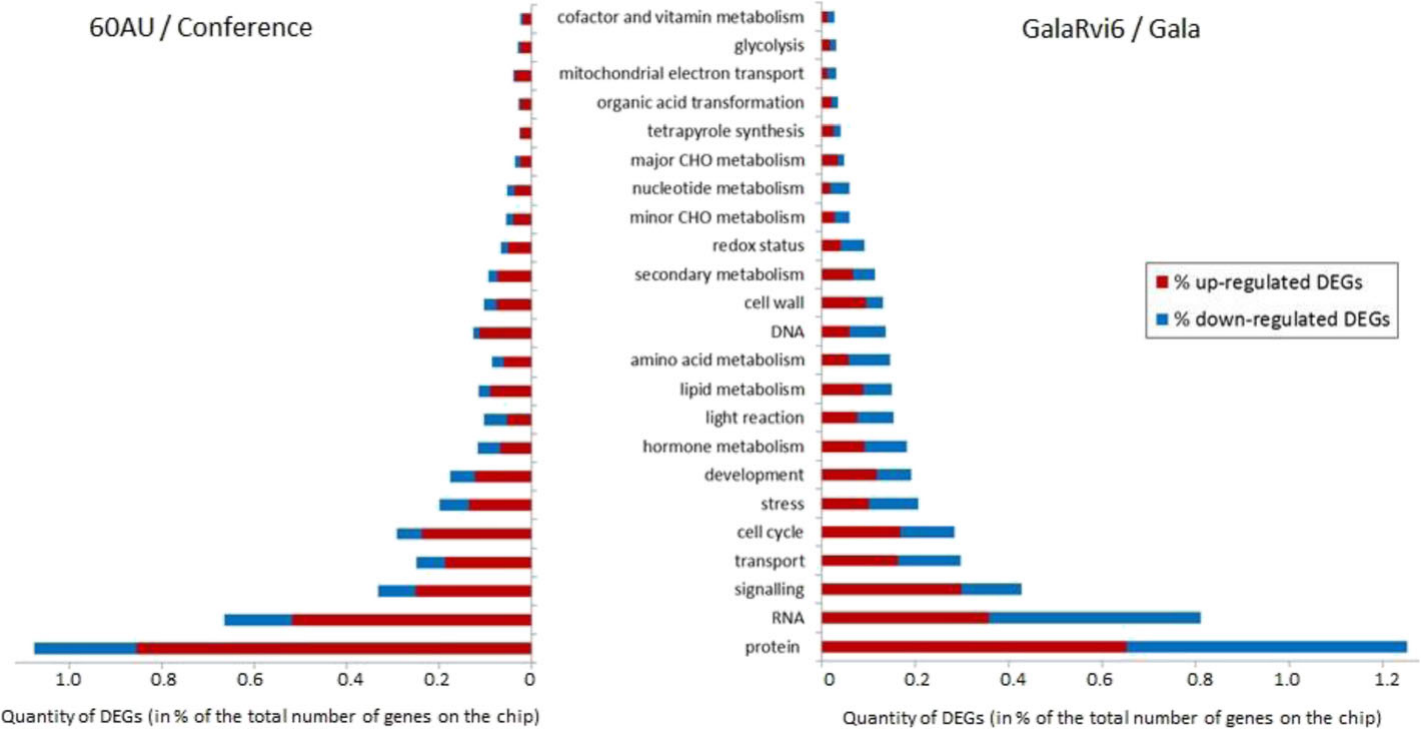
Functional categories of DEGs. Functional classification of pear (60 AU / Conference, on the left) and apple (GalaRvi6 / Gala, on the right) DEGs during their responses to *V. pyrina* and *V. inaequalis* respectively. The number of up- or down-regulated DEGs is expressed as a percentage of the total number of genes present in the Pyrus v1.0 (43,906 genes) and AryANE v2.0 (66,792 genes) microarrays, respectively. DEGs are classified in functional categories according to MapMan 3.5.1R2 bins. Only bins with ≥10 DEGs are presented

In both species, the greatest transcriptomic divergence between the susceptible and the resistant transgenic lines occurred at 8hpi (with 1799 DEGs in apple and 2415 DEGs in pear), with respectively 85 and 83% of these DEGs specifically detected at this time point. Across all time points, the proportion of up-regulated DEGs was higher in pear (76%) than in apple (53%), with the same main functional categories represented: protein metabolism, RNA metabolism, signaling, transport and cell cycle (Fig. 4).

To basically validate the transcriptomic data, 13 DEGS with varied ratios (between − 2.01 and 3.57) have been tested in Q-PCR (Table S2), on the two biological repeats used for transcriptomic analyses. In this study 73 apple DEGs, listed in Table S3, are discussed in the four next sections. Among them about 69% were at 8hpi, QPCR was thus focused on DEGs at that time. The 5 chosen apple DEGs at that time have been selected partly among DEGs discussed below (MYB4, CER4) and partly randomly. Concerning pear, 93 DEGs, listed in Table S3, are discussed in the four next sections. Among them about 89% were at 8 or 24hpi, and induced for a majority (66%). QPCR was then made essentially on DEGs with positive ratios at one of these two times (Table S2). The 8 chosen pear DEGs have been selected partly among DEGs discussed below (DFR, FLS, ACP4, KFB, lacs2) and partly randomly. The QPCR results confirmed the induced or repressed statute of tested DEGs. Because we have not considered the MIQE standards, the significance of these results is limited but we assumed it is sufficient for the purpose presented [25].

#### Specific signaling receptors and pathways between apple and pear

##### Signaling

In the signaling functional category, receptor like kinase (RLK), calcium related DEGs, and small GTP-binding proteins (G-proteins) were predominant in apple and pear. Most of these genes were up-regulated as early as 8hpi.

**Fig. 5:**
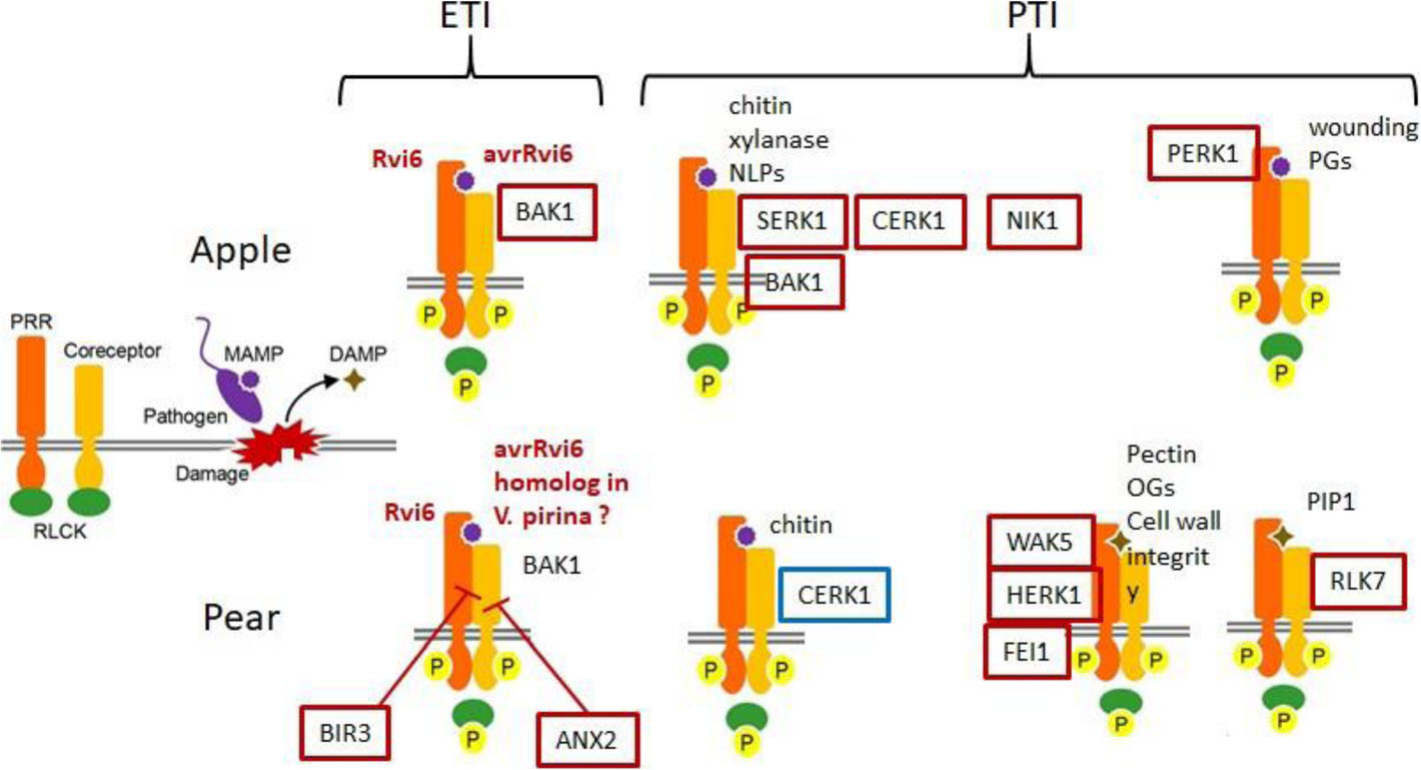
Main complexes of receptors and co-receptors putatively involved in ETI or PTI. Figure adapted from [26]. Main related DEGs activated (in red) or repressed (in blue) in apple (at the top) and in pear (at the bottom). Abbreviations: ANX2: ANXUR2; BAK1: BRI1-associated receptor kinase1; BIR3: BAK1-interacting LRR-RK3; CERK: LysM-RLK chitin receptor kinase; DAMP: damage-associated molecular pattern; FEI1: LRR receptor-like serine/threonine-protein kinase; HERK1: HERCULES1; MAMP: microbe-associated-molecular pattern; NIK1: NSP-interacting kinase1; NLP: necrosis and ethylene-inducing peptide 1-like protein; OG: oligogalacturonides; P: Phosphorylation; PERK1: Proline Extensin-like Receptor kinase1; PIP1: plasma membrane intrinsic protein 1; PG: polygalacturonase; PRR: pattern recognition receptor; RLCK: receptor-like cytoplasmic kinase; SERK1: somatic embryogenesis receptor kinase1; RLK7: Receptor-like kinase7; WAK5: wall-associated kinase 5

Among the 33 and 30 receptor kinases up-regulated, 15 and 20 were RLK with a leucine rich repeat (LRR) domain in apple and pear respectively. Several of these RLKs could putatively be involved in effector-triggered immunity (ETI) or pattern-triggered immunity (PTI) (Fig. 5). For example, *CERK1*, which was up-regulated in apple and down-regulated in pear, has a crucial role in glycan-based microbe-associated molecular pattern (MAMP) perception. CERK1 has recently been shown to be necessary for 1,3-β-D-glucan-triggered immune responses, 1,3-β-D-glucans being important components of fungal and oomycete cell walls. The central role of CERK1 in Arabidopsis immunity is supported by its role in resistance against fungi such as *Alternaria brassicicola*, *Golovinomyces cichoracearum* and *Plectosphaerella cucumerina* [27]. Among the RLKs, PERK1 (a proline extensin-like receptor kinase 1 gene) was up-regulated in apple. Silva and Goring [28] showed that PERK1 may be involved early on in the general perception and response to a pathogen stimulus. Many pattern recognition receptors (PRR) form recognition complexes involving the multitask co-receptors BAK1 and SERK1 [29]. Both co-receptors were also up-regulated in apple. In contrast, a different array of receptors and co-receptors was found up-regulated in pear such as the two negative regulators of BAK1 receptors complex formation (*BIR3* [30] and *ANX2* [31]) and several DAMP receptors such as *RLK7* [32]. Thus *Rvi6*-mediated scab resistance seems to involve a different array of receptors and co-receptors in apple and pear.

Among the calcium related DEGs, calmodulin binding proteins (*IQD13* and *IQD31* in apple; *IQD6* in pear), calmodulin dependent protein kinase (CPK) (*CPK8* and *CPK28* in apple; *CPK1*, *CPK6*, *CPK13* and *CPK21* in pear) and calcium ion binding (*ATCP1*, *ATCBL3* in apple; *CAM3*, *CAM7*, *CRT3* in pear) were up-regulated. Thus, calcium signaling appeared to play a major role in apple as well as in pear during the establishment of *Rvi6*-mediated scab resistance.

The main difference between apple and pear signaling DEGs concerned G-protein known for their role in plant immunity [33]. In apple, only 14 G-protein DEGs were up-regulated (including 2 RABs and 3 ARFs respectively) and 14 DEGs were down-regulated (including 10 RABs). By contrast, most G-protein DEGs were up-regulated in pear (32 out of 36), including NOG1*,* a TRAFAC (translation factors), 13 RABs and 2 ARFs. Some G-proteins are known to interact with Plant U-box type E3 ubiquitin ligases (PUBs), implicated in the regulation of the immune response and cell death [34]. Interestingly we found PUB13 as up-regulated in pear.

##### Hormonal pathways

In the brassinisteroid (BR) pathway, most of the DEGs found were up-regulated in apple and pear. These genes were involved in biosynthesis (*STE1*, *SQE1* and *CYP90A*), in signaling (*SERK1*, *NIK1*, *BAK1* in apple; *BIR3*, *HERK1* in pear) and regulation (*BIM2* (BES1-interacting Myc-like protein), *BES1*/*BZR1* in apple; *BIM2* in pear) of BRs. Anwar et al. [35] showed that BR enhance plant tolerance to biotic and abiotic stresses by activating BES1/ BZR1 transcription factors. BR mediated resistance is known to be independent of SA mediated defense signaling in plants [36]. So, some DEGS seem to indicate that BR signaling is involved in apple and pear scab resistance.

**Fig. 6:**
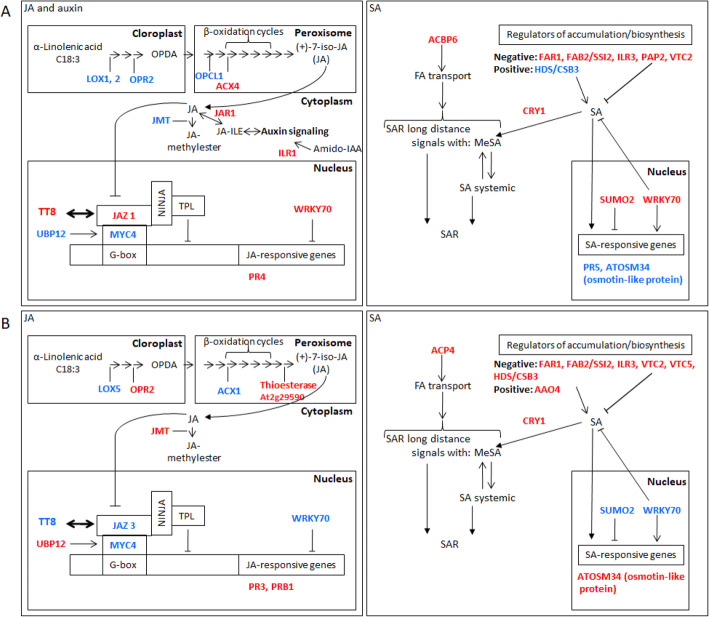
Models of JA, auxin and SA signalings during apple and pear scab host resistance. Apple (**A**) and pear (**B**). In red: up-regulated DEGs; in blue: down-regulated DEGs. Abbreviations: AAO4: aldehyde oxidase 4; ACBP6: acyl-CoA-binding protein; ACP4: acyl carrier protein 4; ACX1, ACX4: acyl-CoA-oxidase; Amido-IAA: amido-indole-3-acetic acid; CRY1: cryptochrome 1; FAB2/SSI2: fatty acid biosynthesis 2/ suppressor of SA insensitive 2; FAR1: FAR-red impaired response 1; G-box: cis-element in the promoter; HDS/CSB3: 4-hydroxy-3-methylbut-2-enyl diphosphate synthase; ILR1, ILR3: indole-3-acetic acid-leucine resistant; JA-ILE: jasmonate-isoleucine; JAR1: jasmonate resistant 1; JAZ: jasmonate-zim domain protein; JMT: jasmonic acid carboxyl methyltransferase; LOX: lipoxygenase; MYC2, MYC4: transcription factor; NINJA: novel interactor of JAZ; OPCL1: OPC-8:0 CoA ligase 1; OPDA: 12-oxophytodienoic acid; OPR2: 12-oxophytodienate reductase 2; PAP2: phytochrome-associated protein 2; PR3, PR4, PR5, ATOSM34/PR5, PRB1;: pathogenesis-related proteins; SUMO2: small ubiquitin-like modifier 2; TPL: TOPLESS co-repressor;; TT8: Transparent Testa 8; UBP12: ubiquitin-specific protease 12; VTC 2, VTC5: vitamin C defective; WRKY: transcription factor

In the auxin pathway, most of the DEGs found were up-regulated in apple and pear. They are involved in biosynthesis (*JAR1* in apple), transport (*PIN1* in apple and pear, *ARG1*, *MDR1*, *AUX1* in apple), signaling (*ILR1* in apple) and regulation by auxin (*IQD13*, *IQD31* in apple, *FQR1* in pear). ILR1 regulates the rate of amido-IAA hydrolysis which results in activation of auxin signaling (Fig. 6A) [37]. *ILR1* transcripts are induced by JA, suggesting that these genes might play roles in JA conjugate hydrolysis or that indoleacetic acid (IAA) release may be JA inducible. JAR1, a jasmonate-amido synthetase active on auxin for adenylation, constitutes another link between auxin and JA signaling [38]. Yet Qi et al. [39] supported the hypothesis that JA and auxin interact positively in regulating plant resistance to necrotrophic pathogens. Thus, in apple, some JA pathway component, interacting positively with auxin signaling, seems activated to promote resistance against *V. inaequalis*.

However, the upstream biosynthesis part of JA pathway (*LOX1*, *LOX2*, *OPCL1, OPR2***)** seems rather repressed in apple (Fig. 6A). Concerning pear, JA biosynthesis part of the pathway seems more positively regulated than in apple, *LOX*5 and *ACX1* were down regulated but *OPR2* and *JMT* were up regulated (Fig. 6B). JMT encodes a JA carboxyl methyl transferase which converts JA into MeJA. Seo et al. [40] proposed that JMT is a key enzyme for the jasmonate-regulated plant responses and that MeJA is the signaling molecule in JA pathway. The signaling pathway depending on JA was also contrasted between apple and pear (Fig. 6 A and B). JAZ proteins are MYC repressors, transcription factors that themselves repress gene expression in response to JA. *JAZ1* was found up-regulated in apple, and thus was a negative regulator of *at4g17880* (MYC4)*,* which was indeed down-regulated. *UBP12* down regulation reinforces the inactivation of MYC4 in apple. UBP12 is known as a stabilizer of another MYC protein: MYC2 [41]. Consistently with the hypothesis of a repressed JA signaling pathway in apple, *WRKY70* (an inhibitor of the JA defense pathway) was up-regulated in that species. Unlike in apple, *JAZ3* was down-regulated in pear. The inhibition of *JAZ3* and the induction of *UBP12* are consistent with the positive regulation of JA in pear. Among JA-responsive genes, the pathogenesis-related PR3, PR4, PR12 act downstream MYC2 activation [42]. In our data, in accordance with the repression of the inhibitor WRKY70, the *PR3* gene was found up-regulated in pear. PR3 encodes a basic chitinase involved in ethylene/jasmonic acid mediated signaling pathway during SAR. PRB1 encodes a basic PR1-like protein and is responsive to ethylene and MeJA [43]. PRB1 was also found induced in pear. To conclude, unlike in apple where only the JA pathway component interacting positively with auxin signaling seems to be activated, the global JA signaling pathway appeared induced in pear.

Concerning the SA pathway, the DEGs observed were mostly negative regulators of SA in pear and in apple and were all activated (Fig. 6A and B). The main difference between apple and pear was the down-regulation of *WRKY70* and *SUMO2* (Small ubiquitin-like modifier) and the activation of OSM34/PR5 in pear, and the opposite in apple. WRKY70 is known as a negative regulator of SA biosynthesis but a positive regulator of SA-mediated defense genes in Arabidopsis [44], among them *PR*5 [45]. SUMO2 acts upstream of SA signaling and suppresses defence signaling in the absence of pathogen [46]. These numerous negative regulators of the SA accumulation/pathway in apple and pear suggest that, even if SAR seems to be engaged (detailed later), it must be independently of SA pathway and in favor of the JA pathway.

The SA and JA/ethylene (ET) pathways are known as mutually inhibitory in many cases and important for immunity against necrotrophic and biotrophic pathogens, respectively [47]. However, Tsuda et al. [48] showed that the loss of signaling flow through the SA pathway can be compensated by another signaling flow through the JA/ET pathways. These findings by Tsuda et al. could explain the absence of SA pathway and the activation of the JA pathway observed in pear *Rvi6* resistance against the hemi-biotrophic fungus *V. pyrina*. BR signaling seems also involved in that resistance. Regarding apple *Rvi6* resistance against the hemi-biotrophic fungus *V. inaequalis*, defenses seem surprisingly deployed independently of the signaling pathways based on the main defense hormones JA and SA, except a JA pathway component interacting positively with auxin signaling, and possibly also thanks to BR signaling.

#### Major role of cell wall-related gene modulation in both species

The plant cell wall is the first contact point during biotic stress and plays an important role in the activation and regulation of defense response strategies. The primary cell wall consists mainly of carbohydrate-based polymers (cellulose, pectin and hemicellulose). The secondary cell wall also contains cellulose, but is enriched in lignin and xylan [27]. The main DEGs detected at 8, 24 or 72 hpi during apple (GalaRvi6 / Gala) and pear (60 AU / Conference) responses to *V. inaequalis* and *V. pyrina*, respectively are listed in Table S4. Our overall results indicate that cell wall genes involved in pectin, cellulose and hemicellulose synthesis and polysaccharide degradation were mostly up-regulated during Rvi6-mediated apple and pear scab resistance, in agreement with many reports on the involvement of most of these genes in the response of plants to pathogens. No callose synthase genes were differentially expressed in our data. The genes related to lignin will be discussed later.

#### Importance of lipid metabolism for cuticle biosynthesis and SAR signaling

##### Cuticle biosynthesis

**Fig. 7:**
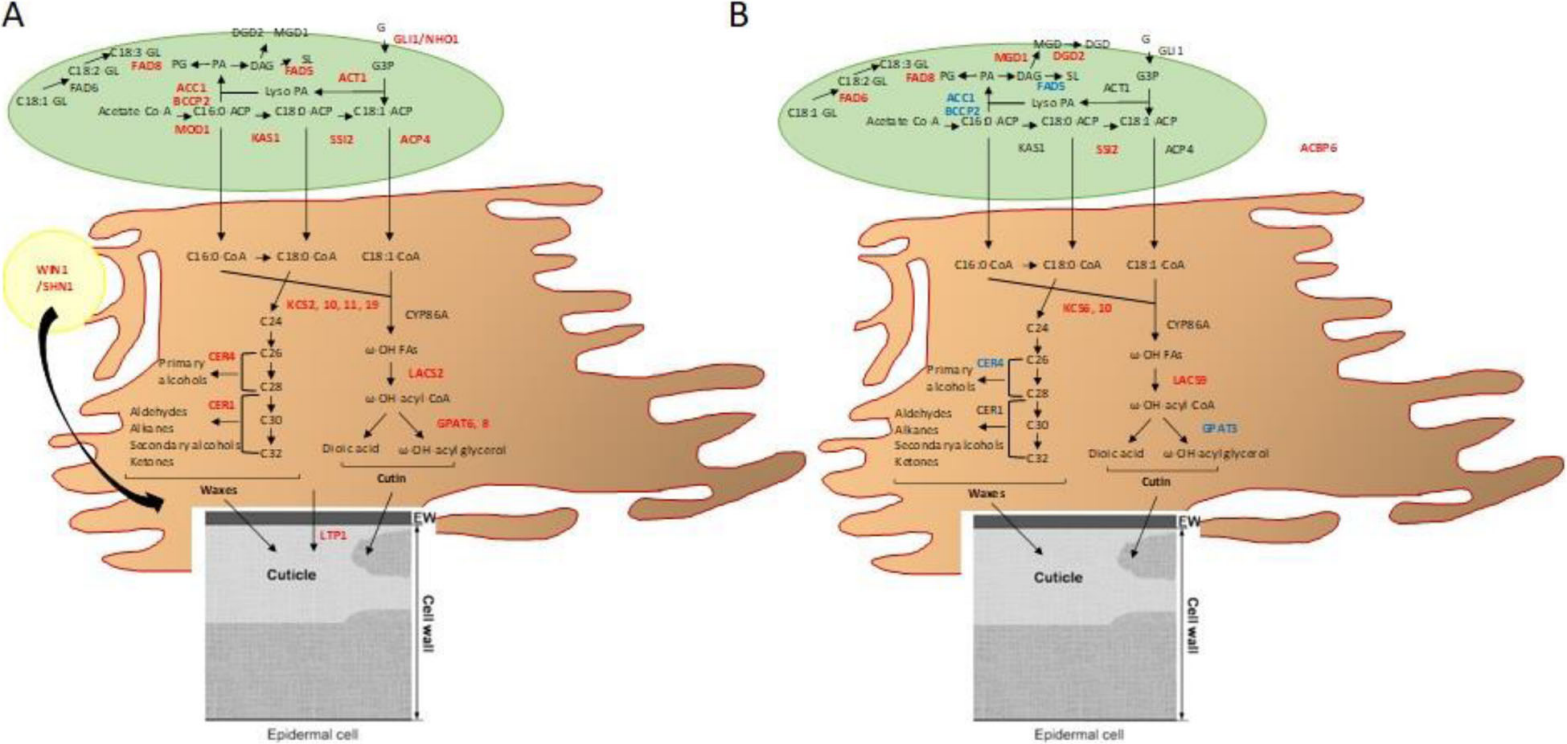
Model of lipid metabolism and cuticle biosynthesis during host resistance of apple and pear. Figure adapted from [50–52]. Pear (**A**) and apple (**B**). In red: up-regulated DEGs; in blue: down-regulated DEGs. Abbreviations: ACBP, acyl CoA binding protein; ACC1, acetyl-CoA carboxylase 1; ACP, acyl carrier protein; ACT1: actin 1; BCCP2, biotin carboxyl carrier protein 2; CER, alcohol-forming fatty acyl-CoA reductase; CoA, coenzyme A; DAG, diacylglycerol; DGDG, digalactosyldiacylglycerol; FAD, fatty acid desaturase; GLI1, glycerol kinase; GPAT, glycerol-3-phosphate acyltransferase; KAS, β-ketoacyl-ACP synthase; KCS, 3-ketoacyl-CoA synthase; LACS, long-chain acyl-coenzyme A synthetase; LTP1, lipid transfer protein 1; MGDG, monogalactosyldiacylglycerol; MOD1, mosaic death 1; PA, phosphatidic acid; PG, phosphatidylglycerol; SL, sulfolipid; SSI2, stearoyl-ACP desaturase; WIN1/SHN1, wax inducer 1/shine 1

A large number of genes involved in lipid metabolism were up-regulated in pear (39 out of 50 DEGs). Most of these up-regulated DEGs were involved in fatty acid (FA) synthesis, like *ACC1* (acetyl-CoA carboxylase 1), *SSI2* (stearoyl-[acyl-carrier-protein] desaturase), *KAS1* (β-ketoacyl-ACP synthase 1), *KCS2* (3-ketoacyl-CoA synthase), *LACS2* (long-chain acyl-coenzyme A synthetase 2). Similarly, many genes involved in lipid degradation were also up-regulated (10 out of 15 DEGs), like *CER4* (alcohol-forming fatty acyl-CoA reductase). One lipid transfer protein (*LTP1*) was also up-regulated. A large number of studies have revealed the role of lipids and lipid metabolites during plant-pathogen interactions, including through the very long chain fatty acid (VLCFA) pathway. These lipids are required for the biosynthesis of the plant cuticle [49]. Throughout the FAs synthesis pathway, several genes were up-regulated in pear leading to synthesis of cuticular waxes and cutin, components of cuticle (Fig. 7A). Cutin and cuticular waxes are known to serve as a physical barrier against pathogens. Several transcription factors (TFs) have been shown to regulate cuticle biosynthesis. The most studied is SHINE1/WAX INDUCER1 (SHN1/WIN1) which is a member of the plant-specific family of AP2/EREBP transcription factors [53]. In our study, *SHN1/WIN1* TF was up-regulated in pear. Non specific Lipid Transfer Proteins (nsLTPs) are known to play a key role in plant resistance to biotic and abiotic stresses and are classified among the PR-14 pathogenesis-related proteins. In our study, *LTP1* was up-regulated in pear. The role of nsLTPs in cutin-monomer transport during cuticle formation has been suggested by Blein et al. [54].

Concerning the FAs and VLCFAs pathways, our results indicate that most of these genes were down-regulated in apple (Fig. 7B). However, unlike in pear, *FAD6*, *MGD1* and *DGD2* were up-regulated. The FAD6 and FAD7/FAD8 enzymes can act on glycerolipids containing either C16 or C18 FAs. Enzymes catalyzing galactolipid biosynthesis are present in the inner [monogalactosyl synthase (MGD)] and outer [digalactosyl synthase (DGD)] membranes of the chloroplast and catalyze the biosynthesis of monogalactosyldiacylglycerol (MGDG) and digalactosyldiacylglycerol (DGDG), respectively. MGDG and DGDG are required for thylakoid formation. Chaturvedi et al. [55] showed that systemic acquired resistance (SAR), but not basal resistance of *Arabidopsis* to *P. syringae* pv. *maculicola*, was affected in the *mgd1* mutant. They suggested that a galactolipid is required for the establishment of SAR.

##### SAR signaling

An intact cuticle is necessary for SAR signal generation and perception [56]. Xia et al. [50] showed that mutations in ACP4, a component of FA biosynthesis, weakens SAR because it affects cuticle formation in the leaf. SAR is also compromised in *lacs2*, *lacs9*, *cer1*, *cer3* and *cer4* mutants. Some of these DEGs were up-regulated in pear (*ACP4*, *LACS2*, *CER1*, *CER4*) and apple (*LACS9*) (Fig. 7). Their results suggest that perception of the mobile signal by the cuticle in distal leaves is as important as its generation at the site of the primary infection, and that an intact cuticle is required for the perception of the mobile SAR signal [50]. They also showed that the acyl CoA binding protein ACBP6 may be involved in the transport of FAs required for the proper development of the plant cuticle as well as the generation of the mobile SAR signal [57]. They suggested that *acbp* plants are unable to generate SAR signal but competent in its perception [50]. *ACBP6* gene was up-regulated in apple, consequently *Rvi6* apple lines could generate the mobile SAR signal (Fig. 6A). Moreover, *ACP4* was up-regulated in pear (Fig. 6B). This suggest that *Rvi6* pear could be competent in the perception of the mobile SAR signal by the cuticle in distal leaves. SAR is also positively regulated by CRY1, upregulated in apple and pear (Fig. 6). Thus, despite the fact that the SA pathway was not activated, apple and pear seem to engage SAR. Indeed, Kachroo and Robin [58] suggested that the accumulation of SA in the distal tissues may not be required for the induction of SAR. Moreover, JA has been suggested to participate in SAR [59] and thus a connection between SAR and JA could be made in pear. Indeed, *PR3* gene was found up-regulated in pear and encodes a basic chitinase involved in ET/JA mediated signaling pathway during SAR. Moreover, exogeneous application of MeJA is known to induce SAR [60]. In pear, via the induction of JMT, MeJA could be produced (Fig. 6B).

Our overall results indicate the importance of lipid metabolism for the two species. In pear, the biosynthesis of cutin and cuticular waxes is increased, which strengthen this barrier against pathogens. In apple, galactolipid biosynthesis seems to be required for the establishment of SAR and thus for resistance to scab. Furthermore, SAR signaling is activated in both species. Whereas the generation of the mobile signal is increased in apple, the perception of this signal in distal tissues is increased in pear, maybe via JA signaling.

#### General activation of the phenylpropanoid pathway and specific metabolite production in apple and pear

The phenylpropanoid pathway is involved in lignin, flavonoid and others metabolites biosynthesis. In our study, genes involved in lignin and flavonoid pathways were up-regulated in pear, 9 out of 9 DEGs and 8 out of 9 DEGs respectively. Most of these genes were expressed as early as 8 h post inoculation. The trend was more towards down-regulation in apple.

##### Repression of the anthocyanin pathway and activation of flavonoid biosynthesis in apple

**Fig. 8:**
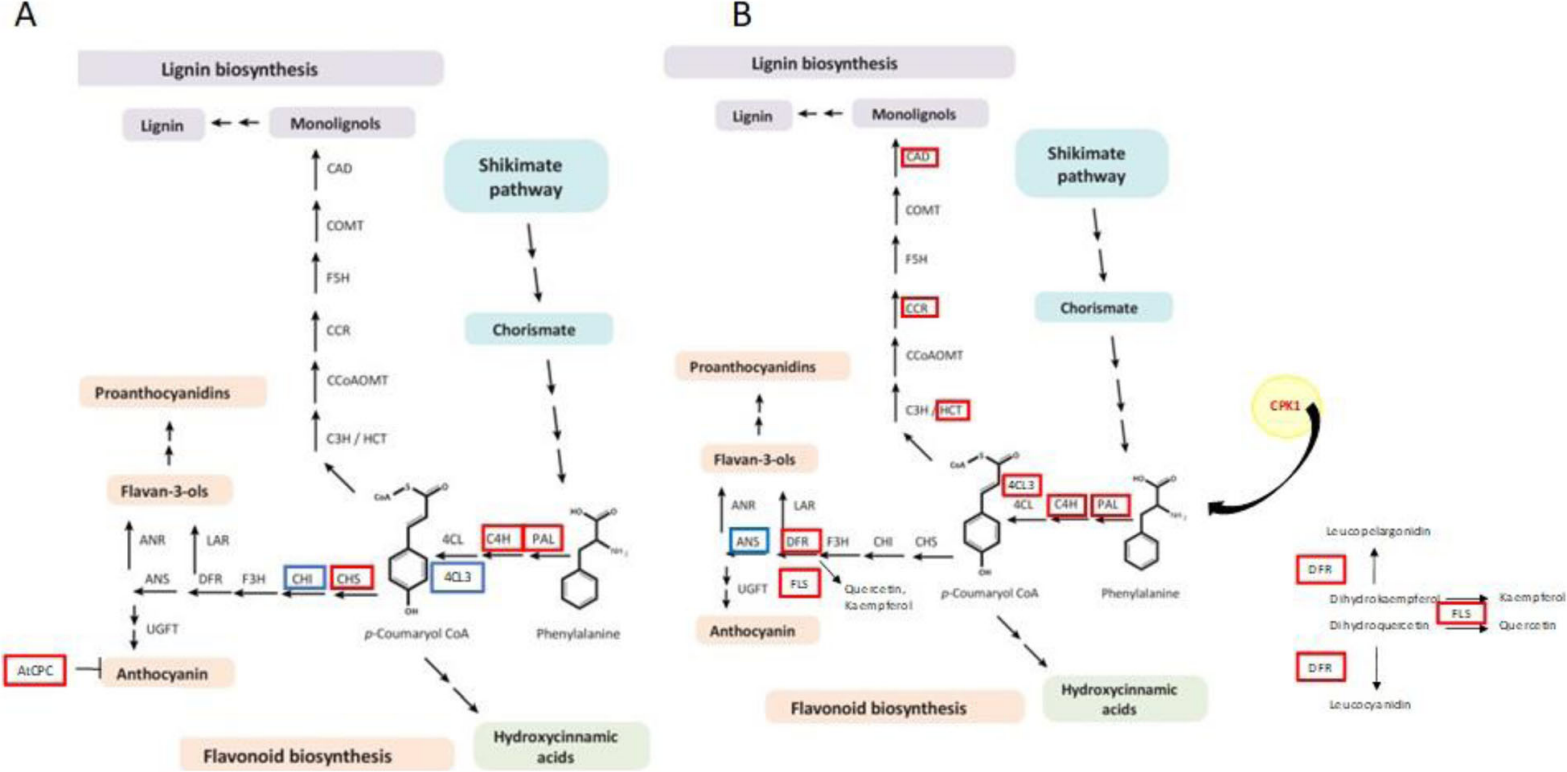
Overview of the phenylpropanoid pathway during host resistance of apple and pear to *Venturia* sp. Figure adapted from [61]. Apple (**A**) and pear (**B**). In red: up-regulated DEGs; in blue: down-regulated DEGs. Abbreviations: 4CL, 4- coumarate-CoA ligase; ANR, anthocyanidin reductase; ANS, anthocyanin synthase; C3H, cinnamate 3-hydroxylase; C4H, cinnamate 4-hydroxylase; CAD, cinnamyl alcohol dehydrogenase; CCoAOMT, caffeoyl-CoA O-methyltransferase; CCR, cinnamoyl-CoA reductase; CHI, chalcone isomerase; CHS, chalcone synthase; COMT, caffeic acid 3-O-methyltransferase; CPC, CAPRICE MYB transcription factor; CPK, calcium-dependent protein kinase; DFR, dihydroflavonol reductase; F3H, flavanone 3-hydroxylase; FLS, flavonol synthase; HCT, hydroxycinnamoyl-CoA shikimate/quinate hydroxycinnamoyl transferase; KFB, Kelch repeat F-box; LAR, leucoanthocyanidin reductase; PAL, phenylalanine ammonia-lyase

In our results, among the apple DEGs, appeared MYB transcription factors regulators of the phenylpropanoid pathway [61]. *At*MYB4 is a MYB repressor of the C4H gene of the lignin pathway. In our study, this gene was down-regulated in apple, therefore it is not repressing the lignin pathway. Indeed, the C4H gene was up-regulated in apple and also in pear (Fig. 8). *At*CPC is a MYB repressor of the anthocyanin pathway [62]. This gene was up-regulated in apple, which suggests that the anthocyanin pathway was therefore not activated (Fig. 8A). Moreover Qi et al. [63] revealed that JAZ proteins interact with bHLH (TT8, GL3, and EGL3) and MYB transcription factors (MYB75 and Glabra1) to repress JA-regulated anthocyanin accumulation and trichome initiation. In our study, *TT8* and *JAZ1* were down regulated in apple (Fig. 6A). Thus, we can assume that the anthocyanin pathway was not activated for apple scab resistance. The flavonoid pathway seems to be engaged because *PAL*, *C4H* and *CHS* were up-regulated in apple. CHS expression leads to the accumulation of flavonoids and isoflavonoids phytoalexins [64]. Treutter stated that flavonoids play a defensive role in apple scab resistance, mentioning that if PAL is inactivated in a resistant cultivar, this leads to strong infection [65].

##### Lignin and flavonoid biosynthesis in pear

In pear, the lignin pathway was activated with many genes up-regulated all along this pathway (Fig. 8 B, Table S4)). The phosphorylation of PAL is accomplished by the activation of *AtCPK1* [66], which was up-regulated in pear in our experiment. The *C4H* and *4CL3* genes were also up-regulated, leading to either flavonoid or lignin pathways. The lignin biosynthesis is sustained in particular with *CAD7* which is involved in the synthesis of lignin precursors. Increased accumulation of lignin can form a barrier against pathogen spread [67].

In pear, we found the flavonoid pathway activated, but without *CHS* up-regulation, which could be explained by the regulation by the SCF E3 ubiquitin ligase complex, involved in protein degradation, and revealed by the up-regulation of ubiquitin-ligase E3s DEGs (and 42 out of 61 in pears). Among the E3-SCF genes, 2 Kelch repeat-containing F-box (KFB) family protein were up-regulated in pear, one of which was highly up-regulated (ratio of 3.16). Another *KFB*-like gene, *at1g23390*, overexpressed in pear, physically interacts with *CHS* and specifically mediates its ubiquitination and degradation [68]. The flavonoid pathway was nevertheless activated, with the up-regulation of dihydroflavonol 4-reductase (DFR) and flavonol synthase (FLS) genes. The FLS gene leads to the biosynthesis of quercetin and kaempferol. These metabolites are activated after priming of tomato seeds with MeJA in response to *Fusarium*, in rust infected leaves of black poplar, are involved in pecan scab resistance, and in Norway spruce rust resistance [69–72]. Therefore, flavonoids, more specifically quercetin and kaempferol, could be involved in pear scab resistance.

Our overall results indicate that most of the flavonoid and lignin genes were up-regulated in pear while as many genes were up-regulated as down-regulated in apple. A general positive correlation between lignin amount and pathogen resistance is observed for several plant-pathogen interactions [73]. Flavonoids seems to be involved in apple scab resistance, as reviewed by Treutter et al. [65]

## Conclusion

**Fig. 9:**
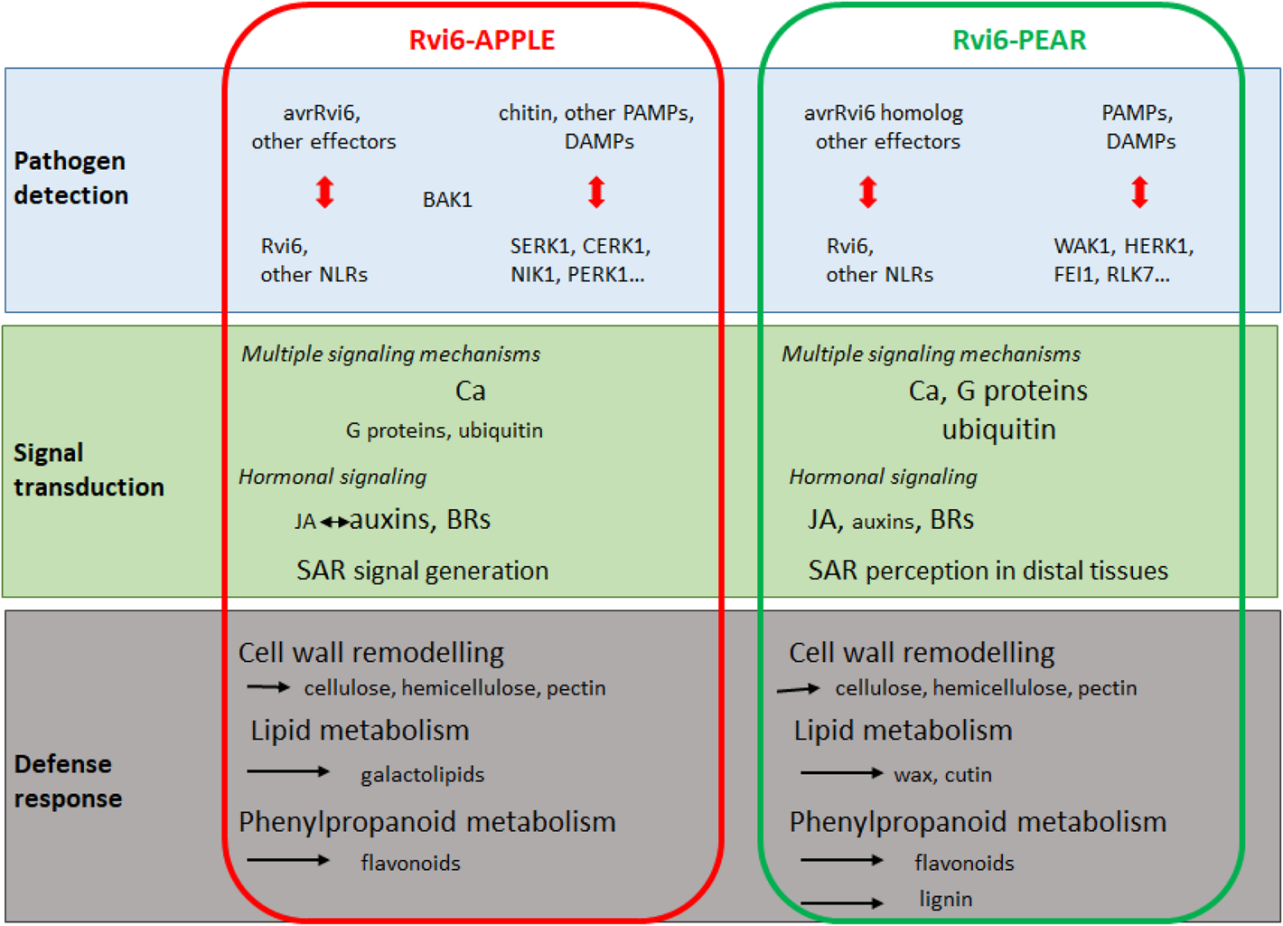
Hypothetical scheme of the main features of *Rvi6*-mediated scab resistance in apple and pear. Based on the most relevant DEGs described in the present study

To conclude, our study allowed elucidating the mechanisms underlying the major gene *Rvi6*-induced resistance in apple, but also in pear. In apple/*V. inaequalis* interaction, once achieved the pathogen recognition thanks to *Rvi6*, signal transduction is triggered by calcium and hormonal signaling, in particular auxin and BRs. This leads to the induction of defense responses such as a slight remodeling of primary and secondary cell wall, galactolipids biosynthesis, the establishment of a SAR and the biosynthesis of flavonoids (Fig. 9). In pear/*V. pyrina* interaction, once achieved the pathogen recognition, signal transduction is triggered by calcium, ubiquitin, G-protein and hormonal signaling involving BRs but especially JA. The perception of a SAR signal in distal tissues is also observed. This leads to the induction of defense responses such as the remodeling of primary and secondary cell wall, the biosynthesis of cutin and cuticular waxes and the biosynthesis of flavonoids and lignin (Fig. 9).

Beyond the precise deciphering of *Rvi6*-induced signaling and defense cascades in apple and pear, this work also revealed that these downstream mechanisms differ between both genera. We can venture that it is also the case at the inter- and intraspecific levels. Comparative transcriptomic analyses of *Rvi6* introgressions in various apple and pear genetic backgrounds would be a great help to test that hypothesis, which has important implications in terms of ideotype design for improved and sustainable resistance. Moreover, the deciphering of the molecular mechanisms underlying varied resistances, quantitative as qualitative, must also be pursued, in order to allow the design of efficient and sustainable host type resistances in apple and pear, allowing adapted genetic background choice, pyramiding and intergeneric transfer strategies. Moreover, whatever the genetic combination used, the adaptation of the pathogen will have to be considered as well.

## Methods

### Biological material

The apple cultivar ‘Gala’ was chosen because of the availability of the transgenic clone P_MdRbc_HcrfVf2–11 (GalaRvi6) already described by Joshi et al. [10] and get from Wageningen UR Plant Breeding.

This transgenic clone expresses the *Rvi6* transgene under the control of the apple *Rubisco* gene promoter and terminator sequences. For this study, it was micropropagated on Murashige and Skoog [74] medium supplemented with 0.5 mg/l 6-benzyladenine and 0.1 mg/l 3-indolebutyric acid. The pear cultivar ‘Conference’ (CF) was chosen for this study because of its high susceptibility to scab [20]. This cultivar was propagated in vitro as previously reported [75].

For pear transformation, the *A. tumefaciens* strain EHA105 [76] containing the ternary plasmid pBRR1MCS-5 with a constitutive *VirG* gene [77] was used. The binary plasmid pMF1-pMdRbc1.6-Vf2-tMdRbc [10] contained the *Rvi6* coding sequence and the regulatory promoter and terminator sequences from the apple *Rubisco* gene.

For apple scab inoculation, the *V. inaequalis* monoconidial isolate used was EU-B04 from the European collection of *V. inaequalis* of the European project Durable Apple Resistance in Europe [78]. For pear scab inoculation, three monoconidial strains of *V. pyrina* were chosen for their aggressiveness on ‘Conference’ (VP98, VP102 and VP137, [20]).

### Production of pear transgenic lines and molecular characterization

Leaves from micropropagated ‘Conference’ plants were used as explants and *Agrobacterium tumefaciens*-mediated transformation was performed according to Mourgues et al. [79]. Transgenic lines and control plants were then propagated in vitro and acclimatized in a greenhouse as described by Faize et al. [80] for apple and Djennane et al. [81] for pear.

The ploidy level of 30 independent pear kanamycin resistant lines was checked by flow cytometry, as described in Chevreau et al. [82], and tetraploid lines were eliminated.

Presence of the transgene and absence of contaminating agrobacteria were monitored by PCR. Genomic DNA extraction from leaves of in vitro shoots was performed according to [83]. Primers used allowed the detection of (i) the specific pMdRbc1.6-Rvi6 fragment, (ii) *A. tumefaciens* presence, (iii) *nptII* gene and (iv) elongation factor 1α (*EF*1*α*). They are listed in Table S5. Amplifications were performed using GoTaq® Flexi DNA Polymerase (Promega) according to the manufacturer’s recommendations. The PCR reaction conditions were identical for the four genes: 95 °C for 5 min, followed by 35 cycles at 95 °C for 30 s, 60 °C for 45 s, 72 °C for 1 min 30s, with a final extension at 72 °C for 5 min. The PCR products were separated on a 1.5% agarose gel.

Expression of the transgenes was assessed quantitatively by QPCR. Total RNA was extracted from leaves of greenhouse-grown plants, according to the same protocol as in “Transcriptomic experiments” section. First-strand cDNA synthesis and QPCR were then performed as described in “QPCR validation of transcriptomic data” section, with 0.3 (*EF1α* and pMdRbc1.6-Rvi6 fragment) of each primer (Table S5) (10 μM) in a final volume of 10 μl. Normalization was done with the reference gene *EF1α* and the non-transgenic genotype ‘Conference’ was used as a calibrator.

The copy number of transgenes was estimated as in [84], by Quantitative PCR (QPCR) on genomic DNA isolated from leaves of micro propagated plants, with the transgene nptII and an endogenous reference gene (actin). Accessions and primer sequences are indicated in Table S5.

### Scab inoculation procedure

Greenhouse growth conditions and mode of inoculum preparation applied in this work were as described in Parisi and Lespinasse [85] for apple and Chevalier et al. [86] for pear. Briefly, the youngest leaf of actively growing shoots was tagged and the plants inoculated with a conidial suspension (2 × 10^5^ conidia ml^− 1^). Symptoms were recorded at 14, 21, 28, 35 and 42 days after inoculation. The type of symptoms was scored using the six class-scale of Chevalier et al. [21] and the quantity of disease was evaluated by the percentage of leaf area with sporulating lesions, recorded on a 7 class-scale as described in Parisi et al. [87].

### Microscopic observations

Two types of microscopic observations were performed. Histological studies were made on samples stained with the fluorophore solophenyl flavine [88]. In brief, leaf discs were rinsed in ethanol 50° before staining in a water solution of solophenyl flavine 7GFE 500 (SIGMA-Aldrich, St Louis USA) 0.1% (v/v) for 10 min. The samples were first rinsed in deionized water, then in glycerol 25% for 10 min. Finally, the leaf samples were mounted on glass-slides in a few drops of glycerol 50%. They were examined with a wide-field epifluorescence microscope BH2-RFC Olympus (Hamburg, D) equipped with the following filter combination: excitation filter 395 nm and emission filter 504 nm.

For cryo-scanning-electronic microscopy (SEM), leaf samples were fixed in glutaraldehyde 4% in phosphate buffer 0.2 M, pH 7.2 under vacuum and stored at 4 °C until observation with a benchtop SEM Phenom G2 Pro (PhenomWorld, Eindhoven, NL).

### Transcriptomic experiments

Leaf samples were immediately frozen in liquid nitrogen and kept at − 80 °C until analysis. Sampling concerned the youngest expanded leaf of each plant labeled the day of the inoculation. Each sample is a pool of leaves from three different plants. Four biological repeats (genotype x treatment x time) were collected during two independent scab inoculation tests. Leaf samples taken just before inoculation (T0) and at 8, 24 and 72 h post inoculation of two biological repeats among these four were then used to perform transcriptomics analyses (Table 2).

**Table 2:** Design of the transcriptomic experiments

Species	Genotype	Scab strain	Type of interaction	Transcriptomic analysis
Pear	60 AU	VP102	R*	R against S at T0^$^, 8, 24 and 72 hpi
	Conference	VP102	S**	R against S at T0, 8, 24 and 72 hpi
Apple	GalaRvi6	VI EUB04	R	R against S at T0, 8, 24 and 72 hpi
	Gala	VI EUB04	S	R against S at T0, 8, 24 and 72 hpi

* *R* resistance, ** *S* susceptibility, ^$^ *T0* sampling time just before inoculation

For RNA extraction, frozen leaves were ground to a fine powder in a ball mill (MM301, Retsch, Hann, Germany). RNA was extracted with the kit NucleoSpin RNA Plant (Macherey Nagel, Düren, Germany) according to the manufacturer’s instructions but with a modification: 4% of PVP40 (4 g for 100 ml) were mixed to the initial lysis buffer RAP before use. Purity and concentration of the samples were assayed with a Nanodrop spectrophotometer ND-1000 (ThermoFisher Scientific, Waltham, MA, USA) and by visualization on agarose gel (1% (weight/volume) agarose, TAE 0.5x, 3% (volume/volume) Midori green). Intron-spanning primers designed on the *EF1α* gene were used to check the absence of genomic DNA contamination by PCR. The PCR reaction conditions were as follows: 95 °C for 5 min, followed by 35 cycles at 95 °C for 30 s, 60 °C for 45 s, 72 °C for 1 min, with a final extension at 72 °C for 5 min. The PCR products were separated on a 2% agarose gel.

Amplifications (aRNAs) were produced with MessageAmpII aRNA Kit (Ambion Invitrogen, Waltham, MA, USA), from 300 ng total RNA. Then 5 μg of each aRNA were retrotranscribed and labelled using a SuperScript II reverse transcriptase (Transcriptase inverse SuperScript™ II kit, Invitrogen, Carlsbad, CA, USA) and fluorescent dyes: either cyanine-3 (Cy3) or cyanine-5 (Cy5) (Interchim, Montluçon, France). Labeled samples (30 pmol each, one with Cy3, the other with Cy5) were combined two by two, depending on the experimental design. For each comparison two biological replicates were analyzed in dye-switch as described in Depuydt et al. [89]. Paired labeled samples were then cohybridized to Agilent microarray AryANE v2.0 (Agilent-070158_IRHS_AryANE-Venise, GPL26767 at GEO: https://www.ncbi.nlm.nih.gov/geo/) for apple, or Pyrus v1.0 (Agilent-078635_IRHS_Pyrus, GPL26768 at GEO) for pear, containing respectively 133,584 (66,792 sense and 66,792 anti-sense probes) and 87,812 (43,906 sense and 43,906 anti-sense probes) 60-mer oligonucleotide probes. The hybridizations were performed as described in Celton, Gaillard et al. [90] using a MS 200 microarray scanner (NimbleGen Roche, Madison, WI, USA).

For microarray analysis we designed two new chips. For apple we used a deduplicated probeset from the AryANE v1.0 ([88]; 118,740 probes with 59,370 in sense and 59,370 in anti-sense) augmented by 14,844 probes (7422 in sense and 7422 in anti-sense) designed on new gene annotations from *Malus domestica* GDDH13 v1.1 (https://iris.angers.inra.fr/gddh13 or https://www.rosaceae.org/species/malus/malus_x_domestica/genome_GDDH13_v1.1). These probes target new coding genes with UTRs when available, manually curated micro-RNA precursors and transposable elements. For transposable elements we used one consensus sequence for each family and a randomly peaked number of elements proportionally to their respective abundance in the genome. The microarray used in this study also have probes for coding genes of *V. inaequalis* but they have not been considered in this study.

For pear the design was done on the *Pyrus communis* Genome v1.0 Draft Assembly & Annotation available on GDR (https://www.rosaceae.org/species/pyrus/pyrus_communis/genome_v1.0) web site. We have downloaded the reference genome and gene predictions fasta files and structural annotation gff file the 21st of September 2015. Using home-made Biopython scripts we have extracted spliced CDS sequences with 60 nucleotides before start and after stop codons to get UTR-like sequences likely to be found on transcripts resulting in a fasta file containing 44,491 sequences. These 60 nucleotides size increase the probability of finding specific probes on genes with high similarity. This file was sent to the eArray Agilent probe design tool (https://earray.chem.agilent.com/earray/) to generate one probe per gene prediction. Options used were: Probe Length: 60, Probe per Target: 1, Probe Orientation: Sense, Design Options: Best Probe Methodology, Design with 3′ Bias. The probeset was then reverse-complemented to generate anti-sense probes and filtered to remove duplicated probes. The final probeset contains 87,812 unique probes targeting 1 (73,612 probes) or more (14,200 probes) potential transcript both in sense and anti-sense.

### QPCR validation of transcriptomic data

In order to validate transcriptomic data, QPCR was performed on a selection of gene/sample associations (Table S2). First-strand cDNA was synthesized using total RNA (2.0 μg) in a volume of 30 μl of 5× buffer, 0.5 μg of oligodT15 primer, 5 μl of dNTPs (2.5 mM each), and 150 units of MMLV RTase (Promega, Madison, WI, USA). The mixture was incubated at 42 °C for 75 min.

QPCR was then performed. Briefly, 2.5 μl of the appropriately diluted samples were mixed with 5 μl of PerfeCTa SYBR Green SuperMix for iQ kit (Quantabio, Beverly, MA, USA) and 0.1 or 0.2 μl of each primer (10 μM) in a final volume of 10 μl. Primers were designed with Primer3Plus or by hand, their volumes were according to their optimal concentration (determined for reaction efficiency near to 100%; calculated as the slope of a standard dilution curve [91];). Accessions, primer sequences and optimal concentrations are indicated in Table S2. The reaction was performed on a CFX Connect Real-Time System (BIO-RAD, Hercules, CA, USA) using the following program: 95 °C, 5 min followed by 40 cycles comprising 95 °C for 3 s, 60 °C for 1 s. Melting curves were performed at the end of each run to check the absence of primer-dimers and nonspecific amplification products. Expression levels were calculated using the ΔΔCT method [92] and were corrected as recommended in Vandesompele et al. [93], with three internal reference genes (GADPH, TUA and ACTIN 7 for apple, GADPH, TUA and EF1α for pear) used for the calculation of a normalization factor. For each couple DEG/sample (sample defining a plant, time, treatment and biological repeat combination), the ratio is gotten by dividing the mean value of CT calculated from 3 technical repeats by the normalization factor obtained for this sample.

### Statistical analyses

For scab inoculation results, the AUDPC based on sporulation scores at 14, 21, 28, 35 and 42 days after inoculation. Statistical analyses were performed with XLSTAT by using the nonparametric Wilcoxon test (*p* < 0.05).

Normalization and statistical analyses performed to get normalized intensity values have been done as in Celton, Gaillard et al. [90]. For each comparison and each probe, we retrieved a ratio of the logarithms of the fluorescence intensities (one per compared sample, cf. Table 2) and an associated *p*-value. The applied p-value threshold to determine DEGs (differentially expressed genes) was 0.05. Through blast analysis, a TAIR accession number (The Arabidopsis Information Resource; https://www.arabidopsis.org/ [94];) has been linked to a majority of apple or pear “probe/corresponding gene” and the couple “TAIR accession/ratio value” has then been used to make a global analysis of functional categories observed in the Mapman software (https://mapman.gabipd.org/homemapman.gabipd.org [95];). The detailed analysis of DEGs has been done through TAIR and KEGG (https://www.genome.jp/kegg/) databases, and bibliography. Metadata for the DEGs discussed in this work are available in Table S3 (Online only).

## Supplementary Information

**Additional File 1: Table S1.** Scab qualitative note of nine transgenic pear lines and non-transgenic Conference inoculated with three *V. pyrina* strains. Percentage of plants in the different classes of symptoms, 42 days after inoculation. **Table S4.** Expression modulation of cell wall related DEGs detected at 8, 24 or 72 h post-inoculation during apple (GalaRvi6 / Gala) and pear (60 AU / Conference) responses to *V. inaequalis* and *V. pyrina*, respectively. In red: up-regulated DEGs, in blue: down-regulated DEGs

**Additional File 2: Table S3.** Metadata for the 73 apple and 93 pear DEGs discussed in this work

**Additional File 3 Table S2.** DEGs analyzed by QPCR. **Table S5**: Accessions and primer sequences for molecular characterization of pear transgenic lines

**Additional File 4: Fig. S1.** Functional analyze of eight pear transgenic lines. Primers are given in Table S5. A) Schematic representation of the T-DNA of the plasmid pMF1, LB & RB: left and right borders, P35S: promoter of the 35S gene of the Cauliflower Mosaic Virus, TNOS: terminator of the nopaline synthase gene of agrobacterium, CODA-NPTII: respectively negative-positive selection genes, REC-LBD: recombinase gene post translationally inducible with a ligand thanks to a ligand biding domain (LBD), RS: recombinase recognition sites, confer [96] for more details about this marker-free plant production system. P1.6MDRBCS: 1600 base pairs (bp) length promoter of the small subunit of the HM222639 *Malus domestica rbc* gene, TMDRBCS: terminator of the small subunit of the HM222639 *Malus domestica rbc* gene, RVI6: coding sequence of the AJ297740 *Malus domestica Rvi6* gene. Location on the T-DNA of a) primers allowing the pMdRbc1.6-Rvi6 633 bp fragment amplification, c) primers allowing the nptII 176 bp fragment amplification, e) primers allowing the Rvi6 131 bp fragment amplification used in QPCR transgene expression determination. B) transgene copy number estimated by QPCR. C) Validation of transgenicity of eight lines by PCR amplifications. Labelling of the molecular ladder is given in kilobase. Primers a) and c) are already detailed in A). b) primers allowing the *agrobacterium tumefaciens* 23S ribosomal RNA 184 bp fragment amplification, d) primers allowing the elongation factor *EF1α* 400 bp fragment amplification. C, S, AK, AM, AO, AS, AT, AU: identification code of the transgenic lines in the series “60”, CF: wild type variety ‘Conference’, N: water as negative control of PCR, P: DNA of agrobacterium strain containing the plasmid PMF1 as a positive control for *in planta* T-DNA elements and agrobacterium presence. **Fig. S2**: Functional categories of DEGs at T0 in apple (GalaRvi6 / Gala, on the left) and pear (60 AU / Conference, on the right). The number of up- or down-regulated DEGs is expressed as a percentage of the total number of genes present in the Pyrus v1.0 (43,906 genes) and AriANE v2.0 (66,792 genes) microarrays, respectively. DEGs are classified in functional categories according to MapMan 3.5.1R2 bins. Only bins with ≥6 DEGs are presented

## Data Availability

The datasets supporting the conclusion of this article are available in the Gene Expression Omnibus (GEO) repository [https://www.ncbi.nlm.nih.gov/geo/] with GSE159179 and GSE159180 accession numbers for apple and pear respectively.

## Abbreviations

AUDPC: area under the disease progress curve
BR: brassinosteroid
CHS: chalcone synthase
CDPK: calcium dependent protein kinase
CRL: cullin ring ligase
DAMP: damage-associated molecular pattern
DEG: differentially expressed gene
DFR: dihydroflavonol 4-reductase
DGDG: digalactosyldiacylglycerol
ET: ethylene
ETI: effector triggered immunity
FA: fatty acid
hpi: hours post inoculation
FLS: flavonol synthase
HR: hypersensitive reaction
IAA: indole acetic acid
JA: jasmonic acid
KFB: Kelch motif containing F-box
LRR: leucine rich repeat
LTP: lipid transfer protein
MAMP: microbe-associated molecular pattern
MeJA: methyl jasmonate
MGDG: monogalactosyldiacylglycerol
nsLTP: non specific lipid transfer protein
NLR: nucleotide binding leucine-rich repeat
PAL: phenyl ammonia lyase
PCR: polymerase chain reaction
PME: pectin methyl esterase
PR: pathogenesis related
PRR: pattern recognition receptor
PTI: pattern triggered immunity
PUB: plant U-box type E3 ubiquitin
Q-PCR: quantitative polymerase chain reaction
QTL: quantitative trait locus
R: resistance
RLK: receptor like kinase
RLP: receptor like protein
RNA-seq: RNA sequencing
SA: salicylic acid
SAR: systemic acquired resistance
SCF: SKP1-CUL1-F box protein
SEM: scanning electronic microscopy
SKP: S phase kinase associated protein
TF: transcription factor
UGT: uridine 5′-diphospho-glucuronyl
VLCFA: very long chain fatty acid
