# Supplementary material for: Successful intergeneric transfer of a major apple scab resistance gene (Rvi6) from apple to pear and precise comparison of the downstream molecular mechanisms of this resistance in both species
Source: BMC Genomics. 2021 Nov 22;22:843. doi: 10.1186/s12864-021-08157-1 (PMC8607633; doi:10.1186/s12864-021-08157-1)
Supplement: Supplementary file 1 — Additional File 1: Table S1. Scab qualitative note of nine transgenic pear lines and non-transgenic Conference inoculated with three V. pyrina strains. Percentage of plants in the different classes of symptoms, 42 days after inoculation. Table S4. Expression modulation of cell wall related DEGs detected at 8, 24 or 72 h post-inoculation during apple (GalaRvi6 / Gala) and pear (60 AU / Conference) responses to V. inaequalis and V. pyrina, respectively. In red: up-regulated DEGs, in blue: down-regulated DEGs [file 12864_2021_8157_MOESM1_ESM.doc]

**Table S1**: Scab qualitative note of nine transgenic pear lines and non-transgenic Conference inoculated with three *V. pirina* strains. Percentage of plants in the different classes of symptoms, 42 days after inoculation.

| Class of symptoms | Conf. | 60C | 60N | 60S | 60AK | 60AO | 60AS | 60AT | 60AU |
| --- | --- | --- | --- | --- | --- | --- | --- | --- | --- |
| Strain VP102 | | | | | | | | | |
| 0 | 0 | 0 | 0 | 0 | 0 | 0 | 0 | 0 | 0 |
| 1 | 0 | 0 | 0 | 0 | 0 | 0 | 0 | 0 | 0 |
| 2 | 0 | 40 | 15 | 41 | 56 | 56 | 88 | 24 | 23 |
| 3a | 0 | 60 | 15 | 59 | 44 | 40 | 12 | 60 | 77 |
| 3b | 0 | 0 | 70 | 0 | 0 | 0 | 0 | 4 | 0 |
| 4 | 100 | 0 | 0 | 0 | 0 | 0 | 0 | 12 | 0 |
| Strain VP132 | | | | | | | | | |
| 0 | 0 | 0 | n.t. | 0 | n.t. | 0 | 0 | 0 | 0 |
| 1 | 0 | 0 | n.t. | 0 | n.t. | 0 | 0 | 0 | 0 |
| 2 | 0 | 75 | n.t. | 58 | n.t. | 67 | 53 | 36 | 37 |
| 3a | 0 | 25 | n.t. | 42 | n.t. | 33 | 47 | 56 | 63 |
| 3b | 0 | 0 | n.t. | 0 | n.t. | 0 | 0 | 8 | 0 |
| 4 | 100 | 0 | n.t. | 0 | n.t. | 0 | 0 | 0 | 0 |
| Strain VP98 | | | | | | | | | |
| 0 | 0 | 0 | n.t. | 0 | n.t. | 0 | 0 | 0 | n.t. |
| 1 | 0 | 0 | n.t. | 0 | n.t. | 0 | 0 | 0 | n.t. |
| 2 | 0 | 31 | n.t. | 10 | n.t. | 9 | 7 | 5 | n.t. |
| 3a | 0 | 44 | n.t. | 52 | n.t. | 32 | 36 | 0 | n.t. |
| 3b | 0 | 13 | n.t. | 33 | n.t. | 27 | 14 | 10 | n.t. |
| 4 | 100 | 12 | n.t. | 5 | n.t. | 32 | 43 | 85 | n.t. |

Class 0: absence of symptoms

Class 1: hypersensitivity (pin-points)

Class 2: resistance (chlorotic lesions, slight necrosis, crinkled aspect)

Class 3a: weak resistance (necrotic or chlorotic lesions with occasional very light sporulation)

Class 3b: weak susceptibility (clearly sporulating chlorotic or necrotic lesions)

Class 4: susceptibility (sporulation only)

**Table S4**: Expression modulation of cell wall related DEGs detected at 8, 24 or 72 hpi during apple (Gala Rvi6 / Gala) and pear (60AU / Conference) responses to *V. inaequalis* and *V. pirina*, respectively. In red: up-regulated DEGs, in blue: down-regulated DEGs.

| Type of cell wall | Cell wall component | DEG name | Function | Expression modulation | |
| --- | --- | --- | --- | --- | --- |
|  |  |  |  | Apple | Pear |
| Primary | Cellulose | SHV3 | cellulose accumulation and pectin linking |  |  |
|  |  | SVL1 | cellulose accumulation and pectin linking |  |  |
|  |  | COB | glycosylphosphatidylinositol-anchored protein |  |  |
|  |  | CESA3 | biosynthesis |  |  |
|  |  | CESA6 | biosynthesis |  |  |
|  |  | CESA9 | biosynthesis |  |  |
| Primary | Hemicellulose: xyloglucans | MUR1 | xyloglucan galactosyltransferase |  |  |
|  |  | MUR2 | fucosyltransferase |  |  |
|  |  | MUR3 | GDP-L-fucose biosynthesis |  |  |
|  |  | XTH33 | xyloglucan/xyloglucosyl transferase |  |  |
|  |  | EXGT-A4 | xyloglucan/xyloglucosyl transferase |  |  |
|  |  | XTR2 | xyloglucan/xyloglucosyl transferase |  |  |
|  |  | XTR7 | loosening the network of cellulose and xyloglucan fibers |  |  |
|  |  | at5g20950 | beta-glucosidase involved in xyloglucan metabolism |  |  |
|  |  | at5g65730 | xyloglucan endotransglucosydase/hydrolase |  |  |
|  |  | at2g14620 | xyloglucan endotransglucosydase/hydrolase |  |  |
|  |  | at3g23730 | xyloglucan endotransglucosylase |  |  |
| Primary | Pectin | GAE1 | pectin biosynthesis |  |  |
|  |  | GAE3 | pectin biosynthesis |  |  |
|  |  | GAE6 | pectin biosynthesis |  |  |
|  |  | at3g16850 | pectinase |  |  |
|  |  | at3g42950 | pectin lyase-like |  |  |
|  |  | at4g23820 | pectin lyase-like |  |  |
|  |  | at3g61490 | pectin lyase-like |  |  |
|  |  | at5g20260 | pectin biosynthesis |  |  |
|  |  | GAUT1 | homogalacturonan biosynthesis |  |  |
|  |  | GAUT8 | homogalacturonan biosynthesis |  |  |
|  |  | at4g19420 | pectinacetylesterase |  |  |
|  |  | at5g26670 | pectinacetylesterase |  |  |
|  |  | at5g09760 | pectinesterase |  |  |
|  |  | at1g23200 | pectinesterase |  |  |
|  |  | ATPMEPCRF | pectin methylesterase PCR fragment F |  |  |
|  |  | ATPME3 | pectinesterase |  |  |
|  |  | PME31 | pectinesterase |  |  |
|  |  | RHM1 | rhamnose biosynthesis |  |  |
|  |  | NRS/ER | nucleotide-rhamnose synthase/epimerase-reductase |  |  |
|  |  | SHV3 | cellulose accumulation and pectin linking |  |  |
|  |  | SVL1 | cellulose accumulation and pectin linking |  |  |
|  |  | at1g70370 | degradation of homogalacturonan |  |  |
|  |  | at1g60590 | polygalacturonase |  |  |
|  |  | at2g43880 | polygalacturonase |  |  |
|  |  | at5g20260 | xylan and pectin biosyynthesis |  |  |
|  |  | AXS2 | rhamnogalacturonan II biosynthesis |  |  |
| Primary | Arabinogalactans | AGP1 | arabinogalactan protein |  |  |
|  |  | AGP18 | arabinogalactan protein |  |  |
|  |  | AGP20 | arabinogalactan protein |  |  |
|  |  | AGP26 | arabinogalactan protein |  |  |
|  |  | AGP17 | arabinogalactan protein |  |  |
|  |  | AGP14 | arabinogalactan protein |  |  |
|  |  | at3g22440 | hydroxyproline-rich glycoprotein family protein |  |  |
|  |  | at3g19020 | leucine-rich repeat family protein (related to HRPG) |  |  |
| Secondary | Cellulose | ATCSLC12 | biosynthesis |  |  |
| Secondary | Hemicellulose: xylans | DET3 | modification of level of wall-bound xylose |  |  |
|  |  | IRX6/COBL4 | modification of level of wall-bound xylose |  |  |
|  |  | IRX9 | family 43 glycosyl transferase |  |  |
|  |  | PGSIP3 | glucuronyltransferase |  |  |
|  |  | KOR1/IRX2 | biosynthesis |  |  |
|  |  | at5g20260 | xylan and pectin biosynthesis |  |  |
| Secondary | Lignin | C4H | biosynthesis |  |  |
|  |  | at5g14700 | biosynthesis |  |  |
|  |  | HCT | cinnamoyl-CoA reductase-related |  |  |
|  |  | OMT1 |  |  |  |
|  |  | 4CL3 |  |  |  |
|  |  | at2g23910 |  |  |  |
|  |  | C4H | cinnamoyl-CoA reductase-related |  |  |
|  |  | PAL1 |  |  |  |
|  |  | PAL2 |  |  |  |
|  |  | UGT72E1 |  |  |  |
|  |  | LAC6 |  |  |  |
