# Supplementary material for: Successful intergeneric transfer of a major apple scab resistance gene (Rvi6) from apple to pear and precise comparison of the downstream molecular mechanisms of this resistance in both species
Source: BMC Genomics. 2021 Nov 22;22:843. doi: 10.1186/s12864-021-08157-1 (PMC8607633; doi:10.1186/s12864-021-08157-1)
Supplement: Supplementary file 4 — Additional File 4: Fig. S1. Functional analyze of eight pear transgenic lines. Primers are given in Table S5. A) Schematic representation of the T-DNA of the plasmid pMF1, LB & RB: left and right borders, P35S: promoter of the 35S gene of the Cauliflower Mosaic Virus, TNOS: terminator of the nopaline synthase gene of agrobacterium, CODA-NPTII: respectively negative-positive selection genes, REC-LBD: recombinase gene post translationally inducible with a ligand thanks to a ligand biding domain (LBD), RS: recombinase recognition sites, confer [96] for more details about this marker-free plant production system. P1.6MDRBCS: 1600 base pairs (bp) length promoter of the small subunit of the HM222639 Malus domestica rbc gene, TMDRBCS: terminator of the small subunit of the HM222639 Malus domestica rbc gene, RVI6: coding sequence of the AJ297740 Malus domestica Rvi6 gene. Location on the T-DNA of a) primers allowing the pMdRbc1.6-Rvi6 633 bp fragment amplification, c) primers allowing the nptII 176 bp fragment amplification, e) primers allowing the Rvi6 131 bp fragment amplification used in QPCR transgene expression determination. B) transgene copy number estimated by QPCR. C) Validation of transgenicity of eight lines by PCR amplifications. Labelling of the molecular ladder is given in kilobase. Primers a) and c) are already detailed in A). b) primers allowing the agrobacterium tumefaciens 23S ribosomal RNA 184 bp fragment amplification, d) primers allowing the elongation factor EF1α 400 bp fragment amplification. C, S, AK, AM, AO, AS, AT, AU: identification code of the transgenic lines in the series “60”, CF: wild type variety ‘Conference’, N: water as negative control of PCR, P: DNA of agrobacterium strain containing the plasmid PMF1 as a positive control for in planta T-DNA elements and agrobacterium presence. Fig. S2: Functional categories of DEGs at T0 in apple (GalaRvi6 / Gala, on the left) and pear (60 AU / Conference, on the right). The number of up- or down-regula [file 12864_2021_8157_MOESM4_ESM.pptx]

## Slide 1
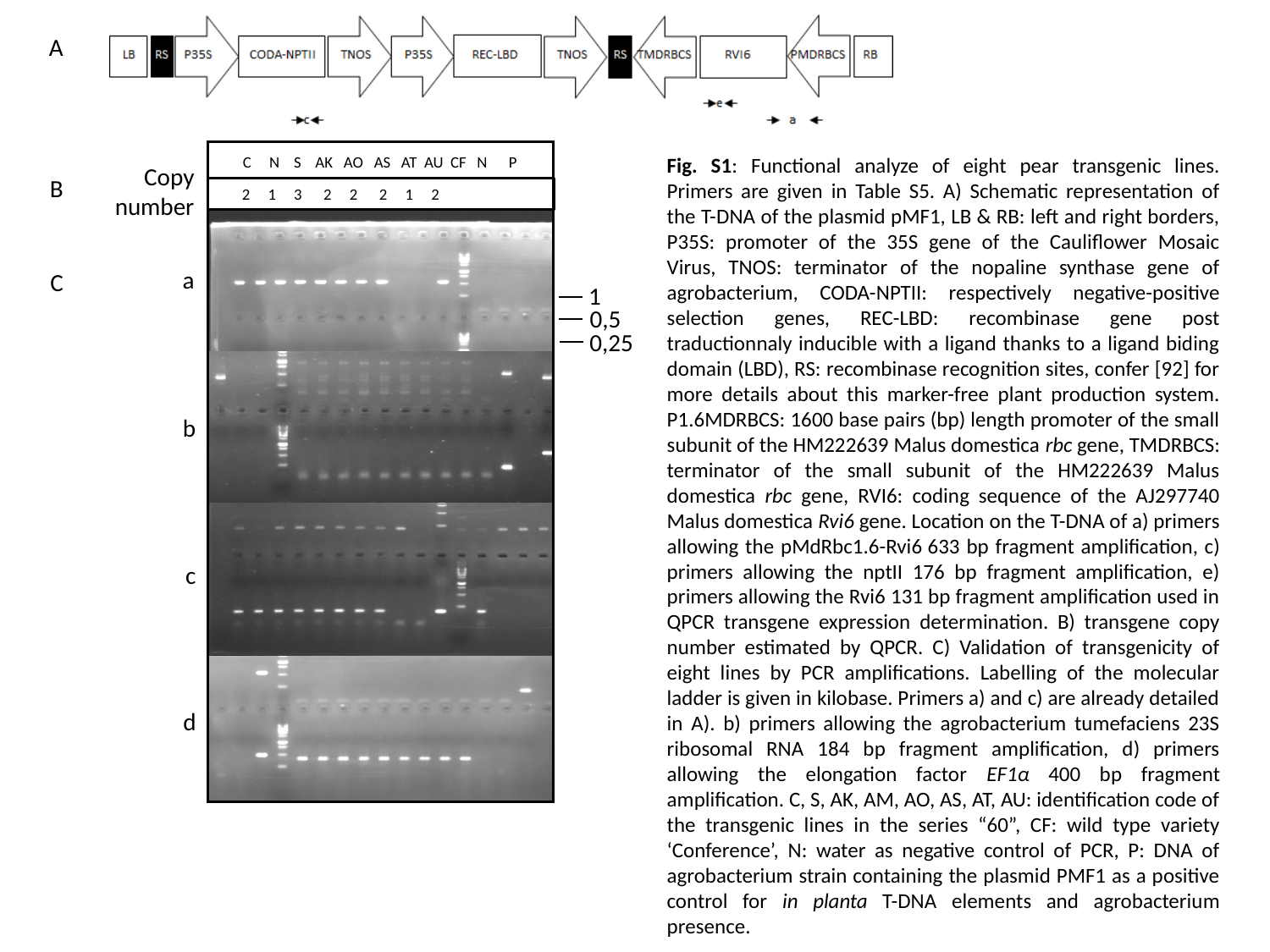

A
 C N S AK AO AS AT AU CF N P
Copy number
B
 2 1 3 2 2 2 1 2
a
C
1
0,5
0,25
b
c
d
Fig. S1: Functional analyze of eight pear transgenic lines. Primers are given in Table S5. A) Schematic representation of the T-DNA of the plasmid pMF1, LB & RB: left and right borders, P35S: promoter of the 35S gene of the Cauliflower Mosaic Virus, TNOS: terminator of the nopaline synthase gene of agrobacterium, CODA-NPTII: respectively negative-positive selection genes, REC-LBD: recombinase gene post traductionnaly inducible with a ligand thanks to a ligand biding domain (LBD), RS: recombinase recognition sites, confer [92] for more details about this marker-free plant production system. P1.6MDRBCS: 1600 base pairs (bp) length promoter of the small subunit of the HM222639 Malus domestica rbc gene, TMDRBCS: terminator of the small subunit of the HM222639 Malus domestica rbc gene, RVI6: coding sequence of the AJ297740 Malus domestica Rvi6 gene. Location on the T-DNA of a) primers allowing the pMdRbc1.6-Rvi6 633 bp fragment amplification, c) primers allowing the nptII 176 bp fragment amplification, e) primers allowing the Rvi6 131 bp fragment amplification used in QPCR transgene expression determination. B) transgene copy number estimated by QPCR. C) Validation of transgenicity of eight lines by PCR amplifications. Labelling of the molecular ladder is given in kilobase. Primers a) and c) are already detailed in A). b) primers allowing the agrobacterium tumefaciens 23S ribosomal RNA 184 bp fragment amplification, d) primers allowing the elongation factor EF1α 400 bp fragment amplification. C, S, AK, AM, AO, AS, AT, AU: identification code of the transgenic lines in the series “60”, CF: wild type variety ‘Conference’, N: water as negative control of PCR, P: DNA of agrobacterium strain containing the plasmid PMF1 as a positive control for in planta T-DNA elements and agrobacterium presence.

## Slide 2
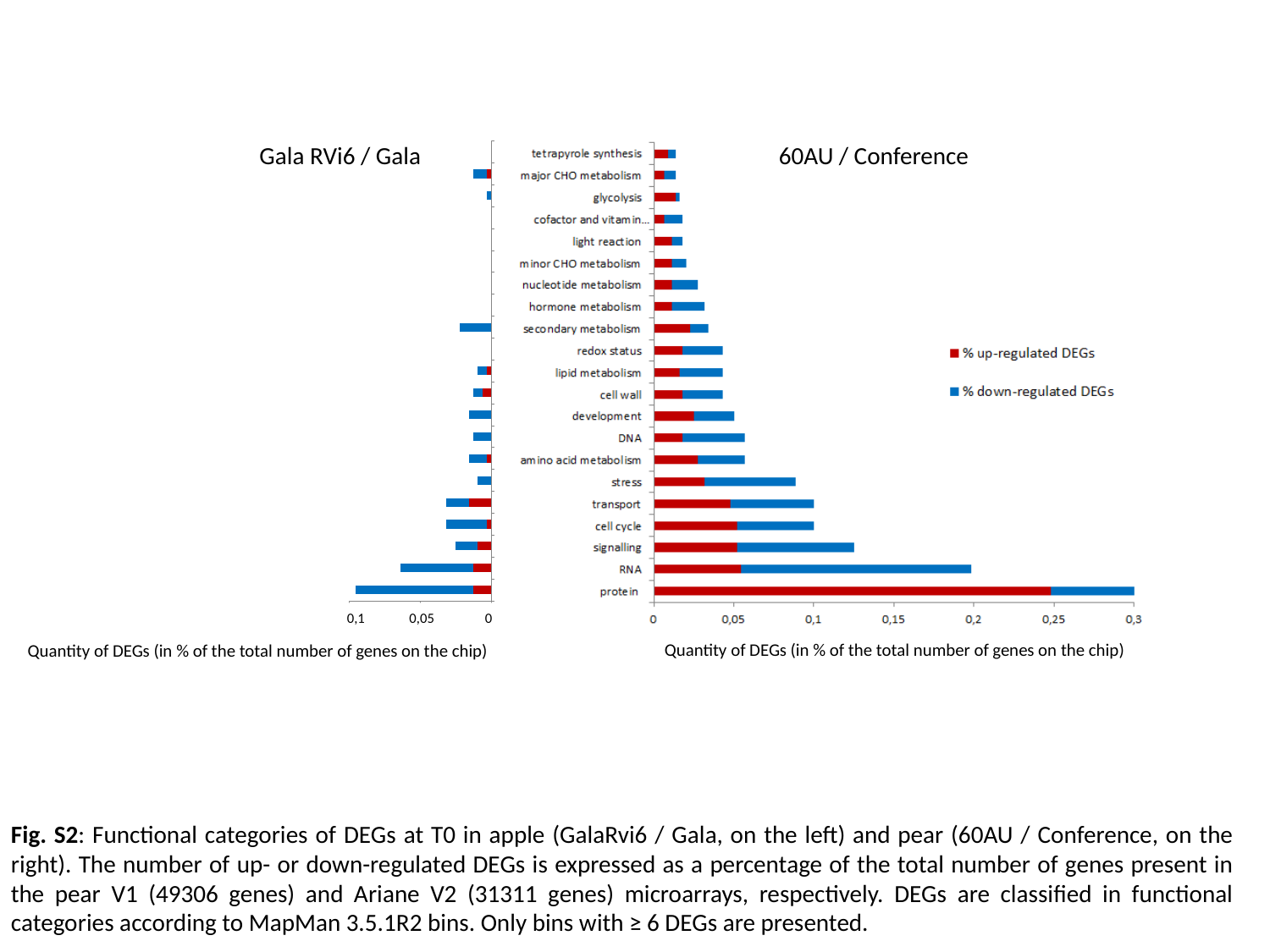

Gala RVi6 / Gala
60AU / Conference
 0,1 0,05 0
Quantity of DEGs (in % of the total number of genes on the chip)
Quantity of DEGs (in % of the total number of genes on the chip)
Fig. S2: Functional categories of DEGs at T0 in apple (GalaRvi6 / Gala, on the left) and pear (60AU / Conference, on the right). The number of up- or down-regulated DEGs is expressed as a percentage of the total number of genes present in the pear V1 (49306 genes) and Ariane V2 (31311 genes) microarrays, respectively. DEGs are classified in functional categories according to MapMan 3.5.1R2 bins. Only bins with ≥ 6 DEGs are presented.
